# Novel Insights Into *Leishmania (Viannia) braziliensis In Vitro* Fitness Guided by Temperature Changes Along With Its Subtilisins and Oligopeptidase B

**DOI:** 10.3389/fcimb.2022.805106

**Published:** 2022-04-21

**Authors:** Anabel Zabala-Peñafiel, Lea Cysne-Finkelstein, Fatima Conceição-Silva, Aline Fagundes, Luciana de Freitas Campos Miranda, Franklin Souza-Silva, Artur A. M. L. Brandt, Geovane Dias-Lopes, Carlos Roberto Alves

**Affiliations:** ^1^ Laboratório de Biologia Molecular e Doenças Endêmicas, Instituto Oswaldo Cruz, Fundação Oswaldo Cruz, Rio de Janeiro, Brazil; ^2^ Laboratório de Imunoparasitologia, Instituto Oswaldo Cruz, Fundação Oswaldo Cruz, Rio de Janeiro, Brazil; ^3^ Laboratório de Pesquisa Clínica e Vigilância em Leishmanioses, Instituto Nacional de Infectologia Evandro Chagas, Fundação Oswaldo Cruz, Rio de Janeiro, Brazil; ^4^ Centro de Desenvolvimento Tecnológico em Saúde, Fundação Oswaldo Cruz, Rio de Janeiro, Brazil; ^5^ Universidade Iguaçu, Dom Rodrigo, Nova Iguaçu, Rio de Janeiro, Brazil; ^6^ Departamento de Computação e Sistemas, Faculdade de Educação Tecnológica do Estado do Rio de Janeiro, Rio de Janeiro, Brazil; ^7^ Departamento de Ciência da Computação, Univeritas-Rio, Rio de Janeiro, Brazil

**Keywords:** *Leishmania (Viannia) braziliensis*, phenotypic characteristics, gene expression, serine proteases, molecular dynamics

## Abstract

Proteases are virulence factors with a recognized impact on the *Leishmania* spp. life cycle. This study considers a set of analyses measuring phenotypic factors of *L. (V.) braziliensis* clinical isolates as promastigotes growth curves, murine peritoneal macrophages infection, inflammatory mediators production, and serine proteases gene expression (subtilisin 13: S13, subtilisin 28: S28, oligopeptidase B: OPB) assessing these isolates’ fitness on *in vitro* conditions. Parasites had different behavior during the early growth phase from day zero to day three, and all isolates reached the stationary growth phase between days four and seven. Macrophages infection showed two tendencies, one of decreased infection rate and number of parasites per macrophage (Infection Index <1000) and another with a constant infection index (≥1400). TNF-α (≥10 pg/mL) detected in infections by 75% of isolates, IL-6 (≥80 pg/mL) by 30% of isolates and low levels of NO (≥0.01µM) in almost all infections. Gene expression showed higher values of S13 (≥2RQ) in the intracellular amastigotes of all the isolates evaluated. On the contrary, S28 expression was low (≤1RQ) in all isolates. OPB expression was different between promastigotes and intracellular amastigotes, being significantly higher (≥2RQ) in the latter form of 58% of the isolates. Predictive structural assays of S13 and OPB were performed to explore temperature influence on gene expression and the encoded proteases. Gene expression data is discussed based on *in silico* predictions of regulatory regions that show plasticity in the linearity index of secondary structures of S13 and OPB 3’-untranslated regions of mRNA, dependent on temperature changes. While hairpin structures suggest an active region of mRNA for both genes above 26°C, pseudoknot structure found in S13 is an indication of a particular profile of this gene at mammalian host temperatures (37°C). Furthermore, the predicted 3D structures are in accordance with the influence of these temperatures on the catalytic site stability of both enzymes, favoring their action over peptide substrates. Data gathered here suggest that *L. (V.) braziliensis* serine proteases can be influenced by the temperature conditions affecting parasite fitness throughout its life cycle.

## Introduction

Leishmaniasis comprises a group of diseases endemic of tropical and subtropical areas that affects approximately 15 million people worldwide causing around 40 000 deaths per year (Alvar et al., 2012). These diseases represent a public health concern due to their geographical expansion and urbanization (World Health Organization (WHO), 2002). They are caused by 20 *Leishmania* spp., which are protozoan parasites transmitted by female sandflies vectors towards mammalian hosts (Akhoundi et al., 2016). Depending on the parasite and vector species involved, as well as host immune status and parasite location in host tissues, three main clinical forms can develop: cutaneous, mucocutaneous and visceral leishmaniasis (Akhoundi et al., 2016).

In the American continent, *Leishmania (Viannia) braziliensis* is one of the main species causing American Tegumentary Leishmaniasis (ATL) characterized by cutaneous localized (CL), mucosal (ML) and, at a lower rate, disseminated (DL) lesions (Zerpa and Ponte-Sucre, 2013). These diverse clinical manifestations are attributed to genetic variability within this parasite, not only in isolates from Brazil but also Argentina and Colombia (Cupolillo et al., 2003; Queiroz et al., 2012; Marlow et al., 2014; Marco et al., 2015; Quaresma et al., 2018; Figueiredo de Sá et al., 2019; Patino et al., 2020). Furthermore, whole genome analysis from public databases showed genomic variability of *L. (V.) braziliensis* from different regions of South America, reinforcing the diversity of natural populations (Patino et al., 2020). Genomic variability translates into phenotypic diversity since *L. (V.). braziliensis* clinical isolates presented distinct patterns of *in vitro* behavior (Fernandes et al., 2016; Rêgo et al., 2018) and great variation of gene expression profiles throughout its life cycle (Adaui et al., 2011a).

In fact, the parasite life cycle may have an important role when determining genetic variability due to the involvement of two or more organisms, the female sandfly vector and reservoir/final mammalian host (Cupolillo et al., 2003). During blood meal, the ingested amastigotes are exposed to temperature (26-27°C) and pH (7.2) changes that triggers transformation and development of motile procyclic, nectonomad, leptomonad, haptomonad and metacyclic promastigotes (Bates, 2007). In the vector, the motile parasite phase develops within the digestive tract, specifically, for *Viannia* species, in the hindgut and midgut (Dostálová and Volf, 2012). Infective metacyclic promastigotes are transferred, during another vector blood meal, to the mammalian host where it is recognized by phagocytes such as neutrophils, dendritic cells and macrophages (Séguin and Descoteaux, 2016). Once internalized in the phagocyte parasitophorous vacuole (PV), promastigotes are challenged by PV increased temperature (32-37°C) and acidic pH (4.5-5.5), conditions that prompt differentiation into non-motile amastigotes. These forms multiply until the host cell collapses and the released amastigotes can either infect other phagocytes or be ingested during another vector blood meal (Gossage et al., 2003; Dostálová and Volf, 2012).

The PV conditions are harsh for the parasites; therefore, their differentiation is an adaptive and survival response modulated by differential gene expression (Clayton, 2002). Various studies, with different *Leishmania* spp., have shown that there are several amastigote-specific up-regulated genes such as amastins, A2, calpain-like cysteine protease, cathepsin L-like cysteine protease, tryparedoxin and tryparedoxin synthetase (Holzer et al., 2006; Depledge et al., 2009; Rochette et al., 2009; Fiebig et al., 2015; Alcolea et al., 2016). The post-transcriptional regulation of these genes has been related to some molecular mechanisms mainly different mRNA 3′untranslated regions and changes in mRNA location (Boucher et al., 2002; Folgueira et al., 2005; McNicoll et al., 2006). Furthermore, it is proposed that mRNAs 2D structures of metalloproteases are more stable than cysteine proteases at 38.4°C (dog skin temperature) and this was correlated with higher mRNA expression of metalloproteases in *L. (L.) infantum* from skin biopsies of asymptomatic, oligosymptomatic and polysymptomatic dogs (Veloso et al., 2020). Interestingly, comparison of the individual effect of increased temperature, acidic pH and combination of both during differentiation showed that the solely influence of increased temperature caused a similar gene expression profile when compared to the combination of both conditions; while acidic pH alone did not prompt parasites differentiation (Alcolea et al., 2010). Altogether, these results highlight the relevance of temperature changes and its effect in gene expression in all *Leishmania* spp. biological stages.

Host invasion and infection establishment are complex processes and *Leishmania* spp., as well as other pathogens, use virulence factors to modulate host-parasite interactions (Matlashewski, 2001). Among these factors, metallo-, cysteine-, aspartic- and serine proteases are the best described in *L. (V.) braziliensis* and correspond to 2.18% of their genome from which 10 to 16% are serine proteases (Silva-Almeida et al., 2014). Serine proteases are classified, according to their structural and functional similarities, in six clans and eight families from which two clans, and respective families, are the most studied in *Leishmania* spp., clan SB-family S8 with subtilisins genes and clan SC-family S9 with oligopeptidase B (OPB) genes (Silva-Almeida et al., 2014; Alves et al., 2018). Subtilisins and OPB were proposed as key enzymes during *Leishmania* spp. life cycle and host-parasite interactions. In fact, subtilisins act as maturases of tryparedoxin peroxidase, the redox-active enzyme of the trypanothione reductase system, involved in parasite survival inside macrophages (Swenerton et al., 2010). Additionally, deletion of OPB in *L. (L.) donovani* parasites changed the macrophage responses to infection with up-regulation of proteins involved in the inflammatory response (Swenerton et al., 2011). Also, OPB is highly expressed in *L. (V.) braziliensis* amastigotes and was proposed as a molecular marker of this stage (Gamboa et al., 2007).

This study focuses on the *in vitro* fitness of *L. (V.) braziliensis* clinical isolates measuring phenotypic factors under simulation of the host microenvironments. Measures such as promastigote growth curves, macrophage infection profiles, inflammatory mediators’ production (TNF-α, IL-6 and NO) and serine proteases expression (subtilisins and OPB) showed heterogenic profiles of these clinical isolates. Additionally, predictive approaches of 3’-untranslated region of mRNA (2D) and protein (3D) structures of both enzymes reinforces the gene expression data obtained here based on temperature variations. Collectively, the findings suggest that *L. (V.) braziliensis* serine proteases expression and structure are influenced by the physicochemical conditions that parasites experience during the life cycle, determining their fitness.

## Materials and Methods

### Parasite Samples and Culture

*L. (V.) braziliensis* clinical isolates (n = 12) were obtained from the biological collection of the Evandro Chagas National Institute of Infectious Diseases (INI – Fundação Oswaldo Cruz), which were previously characterized by multilocus enzyme electrophoresis (Cupolillo et al., 1994) and labelled according to experimental and clinical details as explained in **Supplementary Table 1**. After defrosting, promastigote forms were cultured in Novy-MacNeal-Nicolle (NNN) medium containing 10% of inactivated Fetal Bovine Serum (FBS) at 26°C for four days. Then, the parasites were expanded in Schneider’s insect medium at pH 7.2 supplemented with 10% of inactivated FBS, 200 IU penicillin and 200 mg/mL streptomycin, at 26°C for four days. To obtain each isolate growth curve, promastigotes (5x10^5^/mL) were cultured and maintained in 25 cm^2^ flasks containing 5 mL of Schneider’s insect medium, as described above. Daily, for ten days, an aliquot (10 µL) was taken to determine the number of viable parasites by Neubauer chamber counting.

### *In Vitro* Macrophage Infection

Peritoneal macrophages from female BALB/c mice (4 to 6 week-old) were recovered as described elsewhere (Cysne-Finkelstein et al., 2018), and used for *in vitro* infection assays with stationary phase promastigotes in a ratio of 1:5 (macrophage:parasites). After interaction (37°C, 5% CO_2_, 2h), the culture media was removed by aspiration, and slides were washed with fresh RPMI 1640 media supplemented with 10% FBS. After 24, 48, and 72h of infection, the supernatant of each culture was recovered and stored (-80°C), then, each slide was washed with fresh media, stained through quick panoptical methodology and observed under an optical microscope (Zeiss Primo Star Halogen/LED microscope). A fixed number of 100 macrophages per well was counted to determine the percentage of infected cells and number of parasites inside each one.

### Cytokines Quantification

Macrophage supernatants previously stored (-80°C) were evaluated using ProcartaPlex™ Multiplex immunoassays (Invitrogen, Thermo Fisher Scientific, USA) according to manufacturer instructions. The incubations were performed at room temperature using an orbital shaker. TNF-α and IL-6 levels were determined using Luminex Instrumentation System (Luminex, USA). The values of cytokine levels were expressed as pg/mL.

### Nitric Oxide Quantification

Nitric oxide (NO) production in macrophage supernatants (100 µL) was quantified in 96-well plates using Griess methodology. Absorbance of each well was determined using an Emax ELISA reader (Molecular Devices Inc., USA) at 540 nm. NO concentration was estimated by comparison with NaNO_2_ solution and data were expressed as µM of NO^-3^.

### Primers Design

The set of primers used in this study were designed based on the *L. (V.) braziliensis* Subtilisin 13 (S13 - LbrM.13.0860), Subtilisin 28 (S28 - LbrM.28.2570), Oligopeptidase B (OPB - LbrM.9.0850), Actin housekeeping (LbrM.04.1250) and 40S Ribosomal protein S8 housekeeping gene (LbrM.24.2160) sequences recorded in the GeneDB database (http://www.genedb.org). Subtilisins as well as Actin and 40S Ribosomal protein S8 housekeeping genes were previously designed and analyzed for *L. (V.) braziliensis* (Zabala-Peñafiel et al., 2021). In this study, the primers for OPB were designed using the online software Primer3 v.0.4.0 (http://frodo.wi.mit.edu/primer3/), with all parameters set to default except the product size range, which was adjusted to 80–200 base pairs (bp), and the sequence of this amplicon resulted in sequences compatible with the expected target sequence (**Supplementary Table 2**). All primers sequences and details of standard curves parameters for gene expression are presented in **Supplementary Table 3**.

### RNA Processing and qRT-PCR

Firstly, stationary-phase promastigotes (10^7^ to 10^8^ parasites/mL) and intracellular amastigotes were separately lysed in 1 mL TRIzol containing 200 μL of chloroform. RNeasy Mini Kit (QIAGEN) was used to extract RNA of each sample according to manufacturer instructions. RNA concentrations were measured by spectrophotometry at 260/280 nm and 230/260 nm. DNAse treatment and cDNA synthesis were performed using the SuperScript III Kit (Invitrogen, Thermo Fisher Scientific, USA) with a maximum of 4 µg of total RNA while cDNA concentrations were measured with Qubit ssDNA Assay Kit (Invitrogen, Thermo Fisher Scientific, USA), following the manufacturer’s protocol.

For qRT-PCR assays, 2 μL cDNA (at 1 ng/μL) were used in a final reaction volume of 8 μL, with Power SYBR^®^ Green PCR Master Mix 1X (Thermo Fisher Scientific, USA), 3 μM of forward and 3 μM of reverse primer, in a ViiA7 Real-Time PCR System (Applied Biosystems, Foster City, CA, USA), in 384 well plates. PCR cycling conditions were a first step at 95°C for 10 min, followed by 40 cycles at 95°C for 15 s and 56°C for 1 min. To check for the primers specificity, melting curves were generated after the 40 cycles at 95°C for 15 s, 60°C for 1 min and 95°C for 15 s. Gene expression was calculated by relative quantitation using the comparative Ct method (ΔΔCt), as previously described (Livak and Schmittgen, 2001), with threshold set at 0.04. The housekeeping Actin and protein S8 genes were used as endogenous genes. Gene expression was expressed as fold change (2^-ΔΔCt^), in relation to the sample with the lowest expression for each evaluated gene (Ennes-Vidal et al., 2019). For subtilisins, the reference sample was previously determined (Zabala-Peñafiel et al., 2021) and used for this study; meanwhile, for OPB, all samples (promastigotes and intracellular amastigotes cDNA) were run in order to set the reference sample and analyze differential expression.

### 2D Models of Messenger RNA (mRNA)

Genes sequences for *L. (V.) braziliensis* S13 and OPB were accessed from TriTrypDB server (https://tritrypdb.org/tritrypdb/app) to obtain their respective 5′untranslated region (5′UTR) and 3′untranslated region (3′UTR) sequences. Initially, we identified conserved motifs of the 5’UTR and 3’UTR sequences of S13 and OPB of *L. (V.) braziliensis*, with the orthologous genes from other species (*L. (L.) mexicana, L. (L.) major, L. (L.) infantum, L. (V.) panamensis, L. (L.) donovani*), with the MEME Suite server. Then, the predicted 2D models of the mRNAs were obtained using the *mfold* web server (http://www.unafold.org/) assessing mRNA folding in a temperature gradient (20 to 40°C). These structures were analyzed using single-stranded count (ss-count) achieved by an algorithm to predict the linearity tendency of the structure, whose high values are directly related to the probability to have single strands in the 2D RNA structure.

### Molecular Dynamics

The simulations were performed using NAMD 2.13 (Phillips et al., 2005) with the CHARMMM27 force field (MacKerell et al., 1998). Electrostatic interactions were evaluated using the Particle Mesh Ewald (PME) algorithm with a grid spacing of 1.0 Å. Nonbonded interactions were truncated using a cutoff of 12 Å and a switching function starting at a 10 Å radius. The systems were immersed in orthorhombic boxes using periodic boundary conditions and with a 10 Å layer of water molecules for all coordinates, containing around 9 200 TIP3P water particles (Jorgensen et al., 1998). Na^+^ counterions were added to neutralize the systems. The simulations ran in physiological pH, with the protonation states of the residues selected according to this physiological pH range. Therefore, protonation states were assigned in agreement to the pKa values of their side chains using the psf builder (Humphrey et al., 1996). Equations of motion were integrated using a velocity Verlet integration algorithm with a timestep of 2 fs in an NPT ensemble. The SHAKE algorithm was used to constrain covalent bonds. Energy minimization of the starting structure involved 15 000 steps of the steepest descent method. Next, the system was equilibrated in a two-stage protocol: (i) heating up to the selected temperatures (20°C, 27°C, 37°C and 40°C) by increasing the temperature at every 100 steps, with the CA atom positions restrained using a harmonic potential force constant of 0.25 kcal/mol/Å^2^; (ii) 1 ns of water equilibration at the selected temperature, applying harmonic restraints to the CA atom positions (0.25 kcal/mol/Å^2^ force constant) to allow water molecules to fully envelope the complexes.

For production simulations in the NPT ensemble, the temperature was maintained by using Langevin dynamics, and the pressure was kept constant (1.0 atm) by the Langevin piston method. All residues were free to move and were performed in two rounds for 20 ns each.

The primary amino acid sequences of S13 and OPB from *L. (V.) braziliensis* were submitted to the protein database (PDB) using the basic local alignment search tool (BLAST) and the Hhpred server (https://toolkit.tuebingen.mpg.de/tools/hhpred) to obtain comparative protein structure models. The comparative modeling was built by SWISS-MODEL-Expasy server (https://swissmodel.expasy.org/) using as models the sequence alignment of subtilisin from *Thermococcus kodakaraensis* (3AFG) and OPB from *L. (L.) major* (2XE4). In addition, the sequences were submitted to the PSIPRED server (http://bioinf.cs.ucl.ac.uk/psipred/) to predict the secondary structure. After that, the final model was validated by VERIFY_3D, ERRAT and SAVES scores from the structure analysis (https://servicesn.mbi.ucla.edu/SAVES/).

### Statistical Analysis

To determine significant differences between gene expression levels of each isolate versus each gene reference sample we used 2way ANOVA followed by Dunnett’s multiple comparison test. This test was performed using GraphPad Prism version 9.0.1 for macOS (GraphPad Software, San Diego, California USA, www.graphpad.com).

## Results

### *In Vitro* Infection Profiles

**Figure 1:**
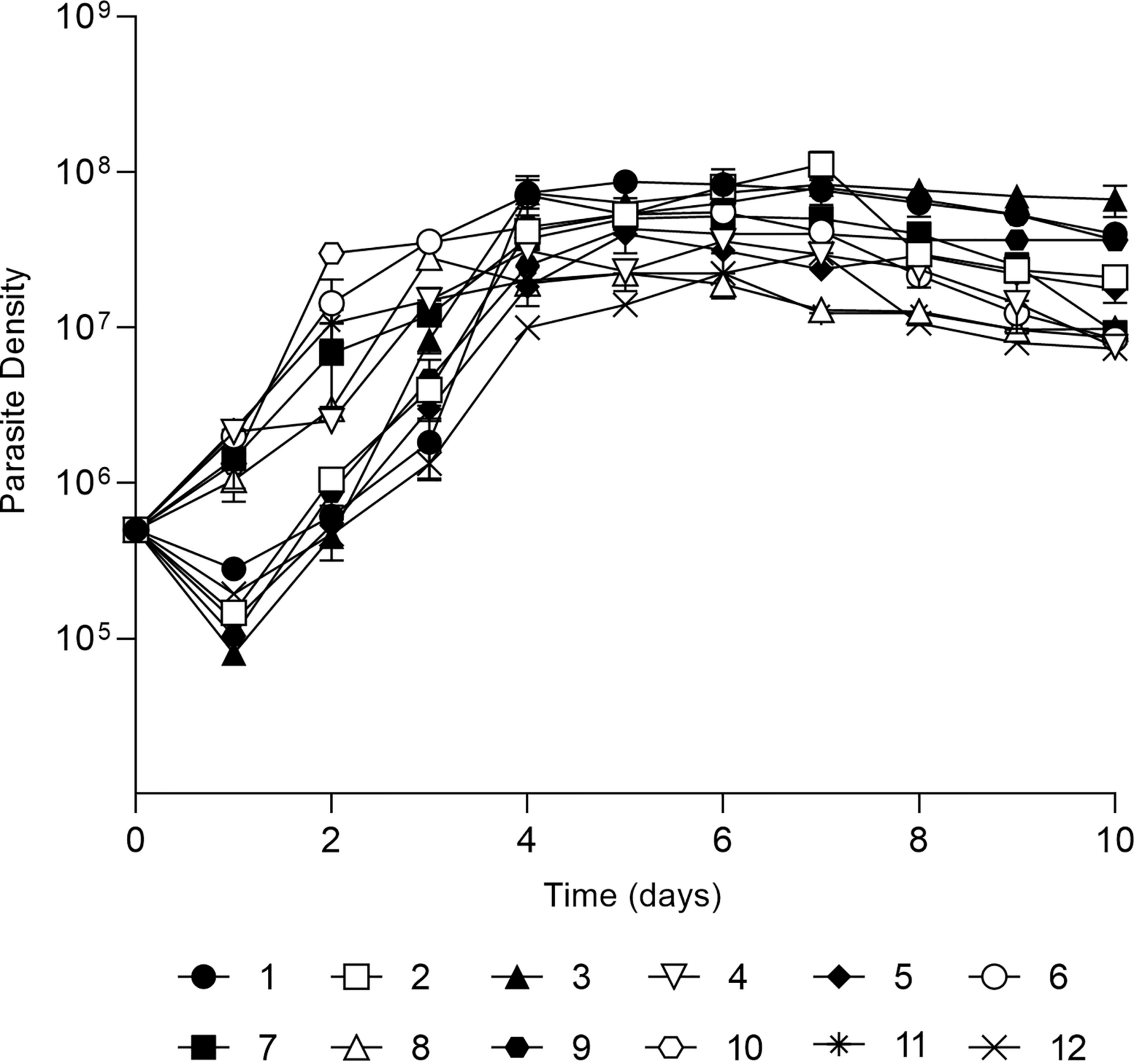
*In vitro* development of 12 *L. (V.) braziliensis* clinical isolates. Growth curve of each isolate in Schneider’s medium, cultures started with 5x10^5^ parasites per mL and growth was followed for 10 days with daily count of viable parasites in a Neubauer chamber. The values are representative of the mean and standard deviation of three independent experiments.

Firstly, *in vitro* growth profiles of each isolate were assessed for ten days showing distinct growth profiles (**Figure 1**). There were two distinct growth profiles during the first 24h, one group of parasites decreased it growth to approximately 1 × 10^5^ parasites/mL while another increased towards 1 × 10^6^ parasites/mL. All parasites grew exponentially until day 4 with a parasite density ranging from 8 × 10^6^ promastigotes/mL to 6 × 10^8^ promastigotes/mL. All isolates reached the stationary phase from day 5 to day 7, with a mean of 2 × 10^7^ promastigotes/mL.

**Figure 2:**
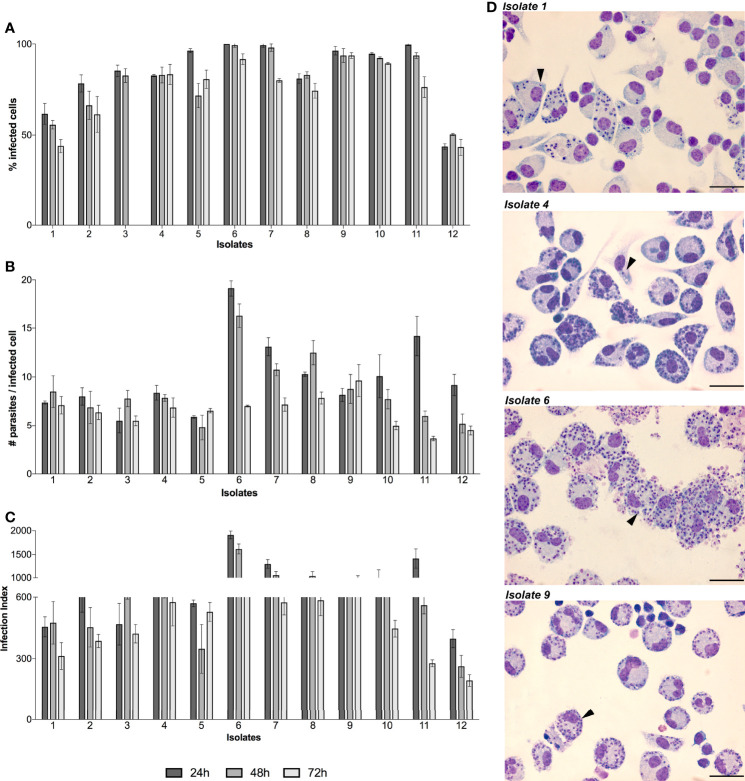
*In vitro* infectivity of *L. (V.) braziliensis* clinical isolates. BALB/c peritoneal macrophages were infected with stationary-phase promastigotes of each isolate (n=12) in a ratio of 1:5 (macrophage:parasites). The infection kinetics was followed by counting and calculating the percentage of infected cells **(A)** and number of parasites inside each infected cell **(B)**. To determine the overall parasite load, the infection index **(C)** was calculated by multiplying individual data from **(A, B)**. The values are representative of the mean and standard deviation of three independent experiments. Light microscopy images of Giemsa-stained macrophages infected by isolates 1, 4, 6 and 9 are showed as representative of all infections, black arrows indicate intracellular amastigotes and scale bars correspond to 100 µm in each image **(D)**.

Then, stationary phase promastigotes were used to infect macrophages and determine infection rates of each isolate and the data showed different infection profiles at 24, 48 and 72h (**Figure 2A**). Exceptionally, isolate 12 presented the lowest infection rate with less than 50% of macrophages infected. Additionally, the number of intracellular amastigotes per macrophage ranged from 5 ± 1.3 (isolate 3) to 19 ± 0.82 (isolate 6) at 24h of infection. These numbers decreased at 48h of infection with values ranging from 4 ± 1.2 (isolate 5) to 16 ± 1.2 (isolate 6) while at 72h of infection it decreased even more ranging from 3 ± 0.2 (isolate 11) to 9 ± 1.6 (isolate 9) (**Figure 2B**). Moreover, the infection index was calculated by multiplying individual data from the percentage of infected macrophages and the number of intracellular amastigotes (**Figure 2C**). This index is differentially high for three isolates at 24 h of infection with values ranging from 1908 ± 82 (isolate 6) to 1413 ± 201 (isolate 11) and 1301 ± 92.7 (isolate 7). At 48h of infection a decreased tendency was seen for all isolates and range from 453 ± 96.5 (isolate 2) to 1614 ± 104.6 (isolate 6) while at 72h of infection there is no value higher than 600. Exceptionally, isolate 12 presented the lowest infection index with 397 ± 44 (24h), 262 ± 53.81 (48h) and 193 ± 28.6 (72h). Additionally, the infection profiles are represented by light microscopy images of four isolates, showing one amastigote per small parasitophorous vacuole. Differences in the number of macrophages infected as well as amastigotes per macrophage can be observed (**Figure 2D**).

### Inflammatory Mediators Production

**Figure 3:**
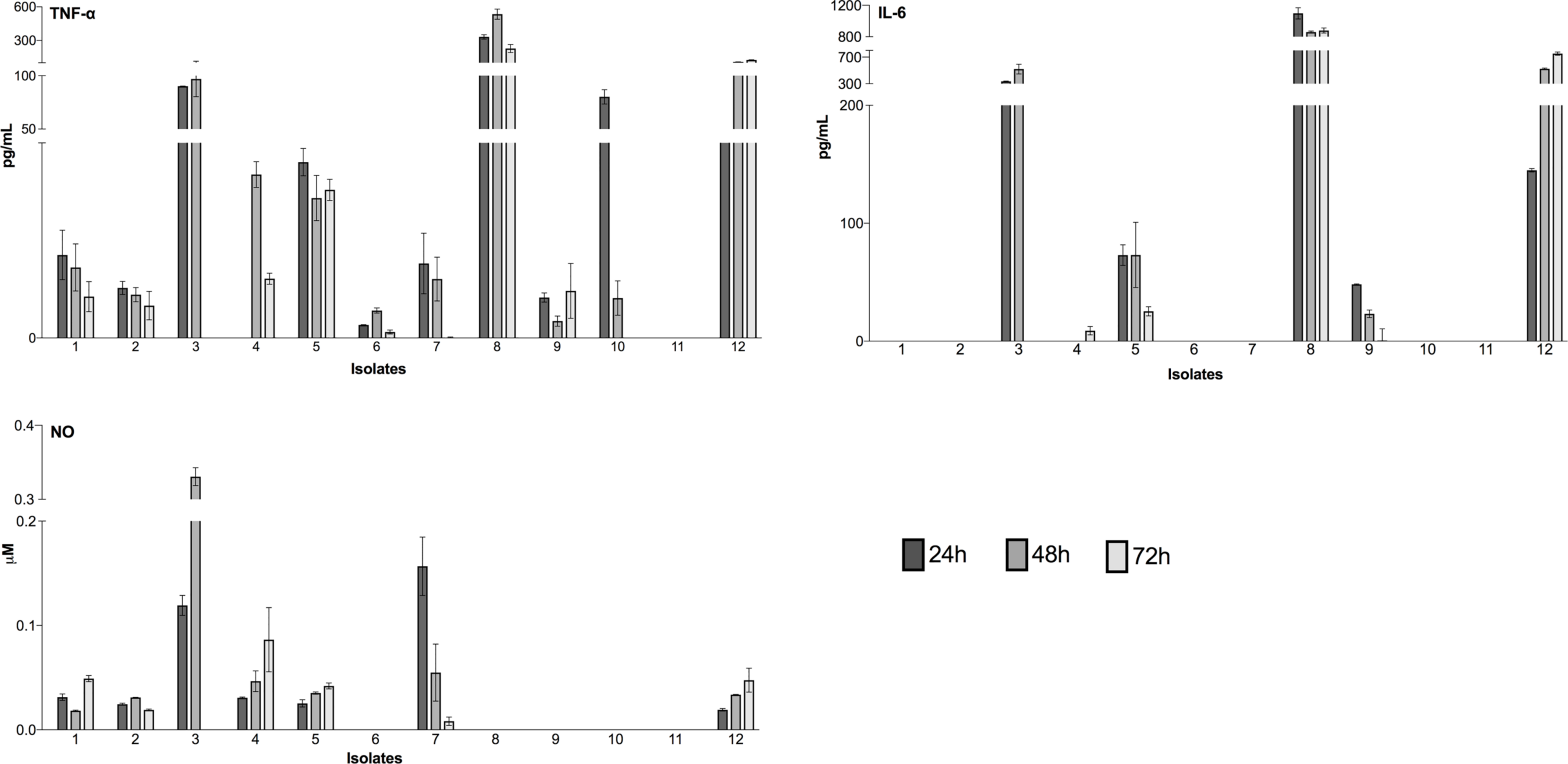
Detection of inflammatory mediators during infection of peritoneal macrophages. Luminex assays detected cytokines (TNF-α and IL-6), and a colorimetric reaction was performed to assess the nitric oxide (NO) concentration. The culture time was assessed at 24h, 48h and 72h. The results represent the differences between the values of infected macrophage culture supernatants and uninfected controls. The data for cytokines (pg/mL) and nitric oxide (µM) concentration are expressed as the means and standard deviation. The values are representative of three independent experiments.

TNF-α, IL-6 and NO production was analyzed from supernatants of each infection at all infection times (**Figure 3**). TNF-α was detected in almost all isolates (except isolate 11). The lowest TNF-α values were detected in infections by isolate 6 varying from 2.651 ± 0.12 pg/mL (24h), 5.61 ± 0.75 pg/mL (48h) and 1.21 ± 0.56 pg/mL (72h). Meanwhile, the highest values by isolate 8 varied from 331.11 ± 27.6 pg/mL (24h), 534.66 ± 63.8 pg/mL (48h) and 227.72 ± 50.5 pg/mL (72h). Contrarily, cytokine IL-6 was detected only in five isolates. The highest values were detected in infections by isolate 8, varying from 1095.89 ± 100 pg/mL (24h), 857.81 ± 18.09 pg/mL (48h) and 877.71 ± 44.9 pg/mL (72h). NO levels were detected in seven isolates, with similar values at 24h of infection, ranging from 0.0019 ± 0.001 µM (isolate 12) to 0.031 ± 0.003 µM (isolate 4). Exceptionally, at 24h, infections with isolate 3 and 7 showed significantly higher values with 0.119 ± 0.01 and 0.123 ± 0.06 µM, respectively. At 48h of infection, a similar pattern was detected only with isolate 3 showing the highest value of NO 0.331 ± 0.01 µM. Meanwhile, at 72h of infection the detected levels were low and very similar except for isolate 4 with 0.086 ± 0.03 µM.

### Serine Proteases Differential Expression

**Figure 4:**
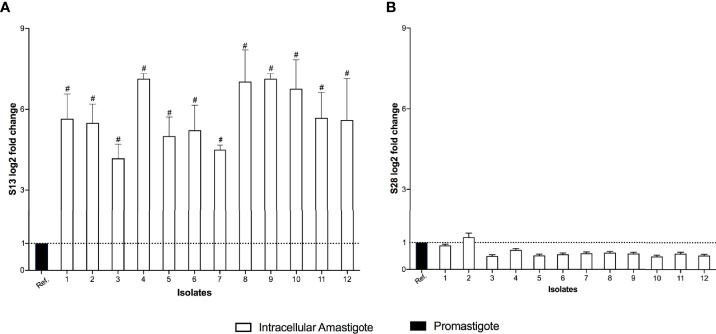
Subtilisins differential gene expression. The real-time quantitative PCR results show the relative quantification values of subtilisin 13 (S13 log2 fold change) **(A)** and subtilisin 28 (S28 log2 fold change) **(B)**. Actin and protein S8 were used as endogenous controls. The ΔΔCt value of each subtilisin gene was calculated pair-to-pair between the reference sample (Ref.) and intracellular amastigotes. The dashed line indicates the reference sample relative quantification level = 1. The graph presents the mean of two independent experiments performed in triplicate. Statistical difference is represented by ^#^ *p* ≤ 0.0005.

**Figure 5:**
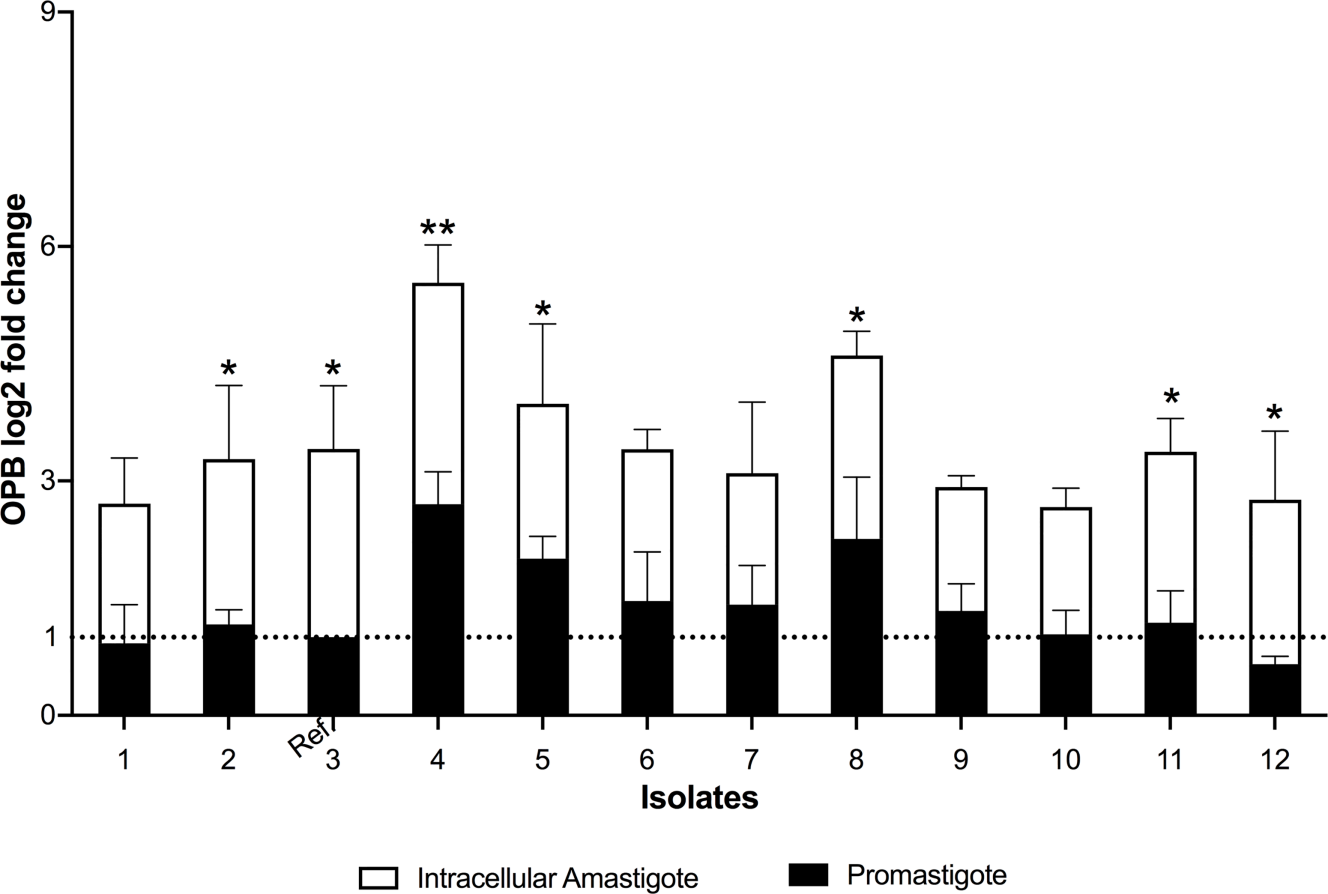
Oligopeptidase B differential gene expression. The real-time quantitative PCR results show the relative quantification values of oligopeptidase B (OPB log2 fold change). Actin and protein S8 were used as endogenous controls. The ΔΔCt value of OPB gene was calculated pair-to-pair between promastigotes and intracellular amastigotes, and the promastigote sample with lowest expression was set as the reference sample (promastigote form of isolate 3, Ref.). The dashed line indicates the reference sample relative quantification level = 1. The graph presents the mean of two independent experiments performed in triplicate. Statistical difference is represented by *p ≤ 0.05 and **p ≤ 0.005.

The differential expression of S13 and OPB were evaluated in 12 clinical isolates of *L. (V.) braziliensis.* S13 gene expression was significantly higher in intracellular amastigotes of all isolates (**Figure 4A**), while S28 remained at basal levels (**Figure 4B**). Moreover, analysis of OPB gene expression was based on the fold difference in gene expression compared to the promastigote form isolate 3 (Relative Quantification = 1), defined as the reference sample. The results showed that OPB gene expression was significantly higher in 58% of the analyzed intracellular amastigotes samples (isolates 2, 3, 4, 5, 8, 11 and 12) as well as in promastigotes from isolates 4 and 8 (**Figure 5**). It is important to mention that negative controls (macrophages without infection) were run and, as expected, the Relative Quantification (RQ) values for each target gene were very low since the designed primers are specific for *Leishmania* sp. RQ values were S13 = 0.072, S28 = 0.025 and OPB = 0.098, this data is not shown in the figures.

### Secondary Structure of Serine Proteases mRNA

**Figure 6:**
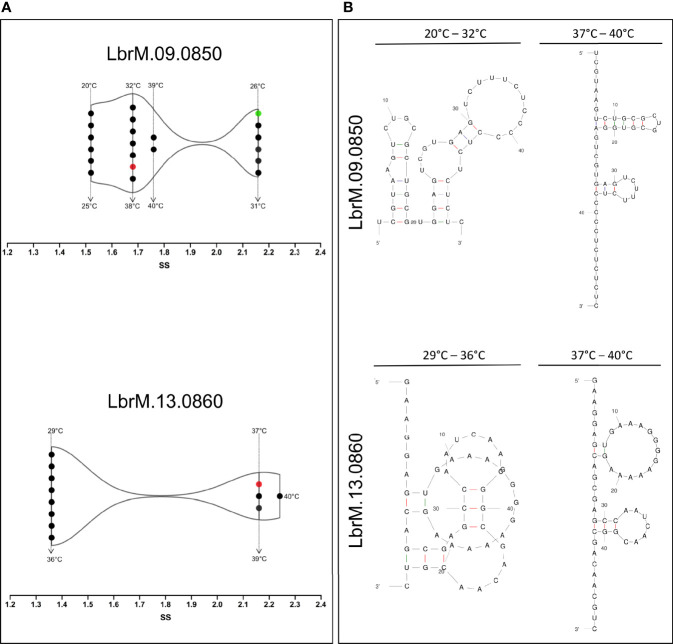
Temperature kinetics of secondary structure modulation of 3’-untranslated region of mRNA sequences of *L. (V.) braziliensis*. **(A)** The temperature-based modulation event was done by single-stranded (SS) frequency measures of the OPB (LbrM.09.0850) and S13 (LbrM.13.0860) gene 3’-UTR of mRNA sequences. The single-stranded structures (ss) were assessed at different temperatures (•). The red symbol represents the mammalian temperature at 37°C and in green the sand fly vector temperature at 26°C. **(B)** Different types of 3’-UTR of mRNA sequences secondary structures, representative of the modulation events in different temperature ranges.

The single-stranded frequency of the OPB and S13 3’UTR and 5’UTR sequences was evaluated. Predictive analysis indicated that temperature variation only influenced the structural change in the 3’UTR regions (**Figure 6A**). The OPB 3´UTR ss-count values varied in four different temperatures ranges: 1.5 ss from 20 to 25°C (Δg -11.66 ± 0.7 kcal/mol), 2.1 ss from 26 to 31°C (Δg = -9.36 ± 0.7 kcal/mol), 1.7 ss from 32 to 38°C (Δg = -7.06 ± 0.6 kcal/mol) and 1.8 ss from 39 to 40°C (Δg = -5.86 ± 0.2 kcal/mol). Also, S13 3´UTR in three temperature ranges: 1.3 ss from 29 to 36°C (Δg = -6.92 ± 0.7 kcal/mol), 2.1 ss from 37 to 39°C (Δg = -5.39 ± 0,2 kcal/mol) and 2.4 ss at 40°C (Δg = -4.97 kcal/mol) (**Figure 6A**). These values indicate the probability of a unique chaining of each base in all the calculated folds for OPB mRNA at 26 to 31°C. Meanwhile, for S13 mRNA, this event occurs in a higher temperature range, from 37 to 39°C. In particular, it is noteworthy that S13 presents values of ss-count only from 29°C. However, at 20 to 28°C it showed Δg -9.45 ± 0.8.

These analyses allowed us to explore the secondary structure types of OPB and S13 3’UTR regions that are formed from temperature variation (**Figure 6B**). Prediction of OPB 3’UTR region at 20 to 32°C showed a multi-branch loop and at 33 to 40°C showed a double hairpin loop predominance. The large hairpin loop showed six paired and three unpaired bases, and the minor one showed three paired and five unpaired bases. Concerning the prediction of S13 3’UTR region at 29 to 36°C, it showed a pseudoknot structure type which is one of the most common types in which the unpaired base of two hairpin structures. The 37 to 40°C track showed a double hairpin loop predominance, as a large hairpin loop of two paired and thirteen unpaired bases and the minor of three paired and six unpaired bases (**Figure 6B**).

### 3D Arrangement of Serine Proteases

**Figure 7:**
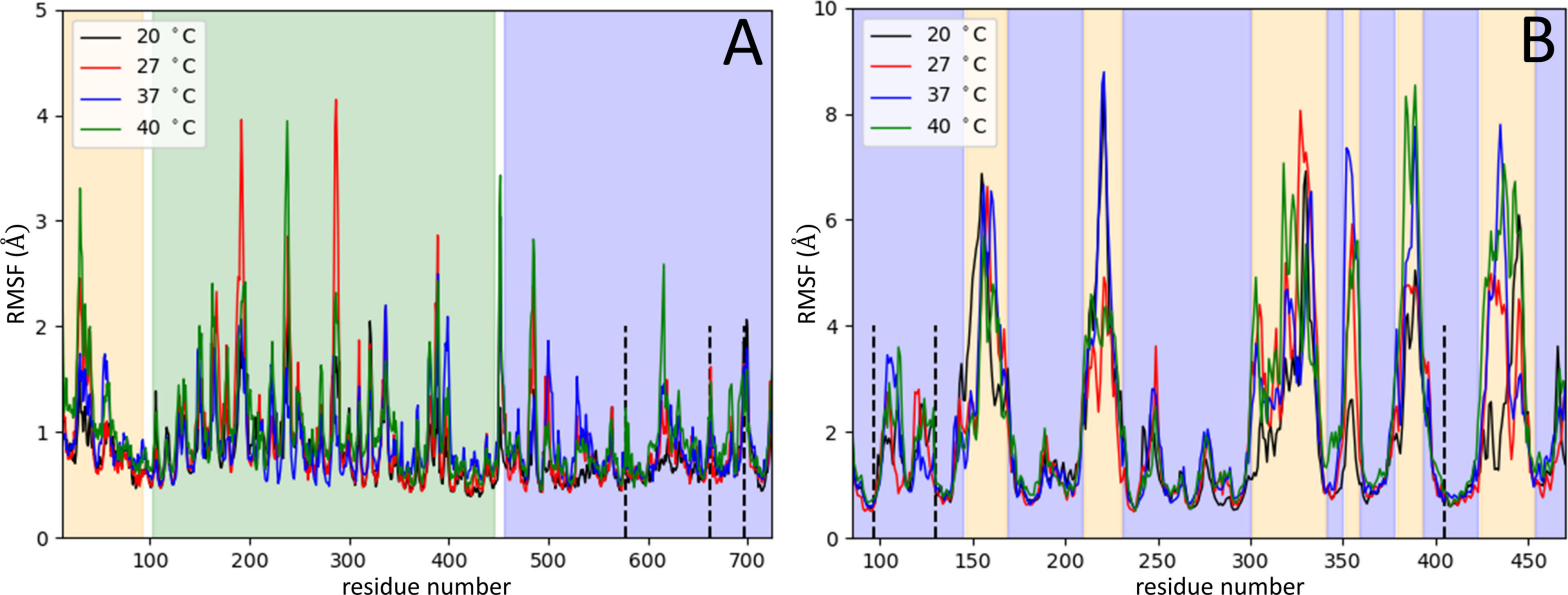
Serine proteases variation in Root Mean Square Fluctuation. The RMSF measures (Å) were obtained per residue at different temperatures: 20°C (black), 27°C (red), 37°C (blue) and 40°C (green). RMSF variation for residues of OPB **(A)** and for residues of S13 **(B)**. In panel **(A)**, the OPB catalytic domain N-terminal region (pale orange), the C-terminal region (blue), the β-propeller domain (green), the hinge regions (white), and the catalytic triad (Ser577, His697, and Asp662) in dotted lines. Panel **(B)** represents the catalytic domains of S13 (blue region), in which the 6 six regions with the highest flexibility (pale orange), and the catalytic triad (Ser-577, His-697, Asp-662) in dotted lines.

In the first stage of these assays, the Root Mean Square Deviation values (RMSD) were assessed to show both protein structures equilibration (S13 and OPB) before the production simulations (**Supplementary Figure 1**), as well as to indicate if the structures of the enzymes remained stable at different temperatures during production simulations. These analyses showed that such enzymes can occur in the respective favorable conformations for enzymatic catalysis. Thus, RMSD values and native contacts reflect the stability of the enzymes over the simulations at different temperatures. Therefore, the rise in temperature does not cause a destabilization or an unfolding of both enzyme structures. After that, the enzyme’s intermolecular interaction, which occurred at the beginning of the simulations, was explored by quantifying native contacts of interactions during the simulation trajectories which is an additional indicator of enzyme stability (**Supplementary Figure 2**). The results showed the enzymes maintained more than 95% of their initial contacts, demonstrating their structural stability during the production run. Then, the Root Mean Square Fluctuation (RMSF) was calculated for CA´s atoms on the ensemble trajectories permitting to assess per-residue flexibility profiles and calculate the fluctuation degree of every residue of S13 and OPB catalytic domains. RMSF was used to better understand the structural flexibility of both enzymes. These simulations were performed at different temperatures to compare the RMSFs degree of similarity. The atomic fluctuations for CA´s atoms were calculated from 20 ns of MD simulations at each temperature: 20°C, 27°C, 37°C and 40°C (**Figures 7A, B**).

**Figure 8:**
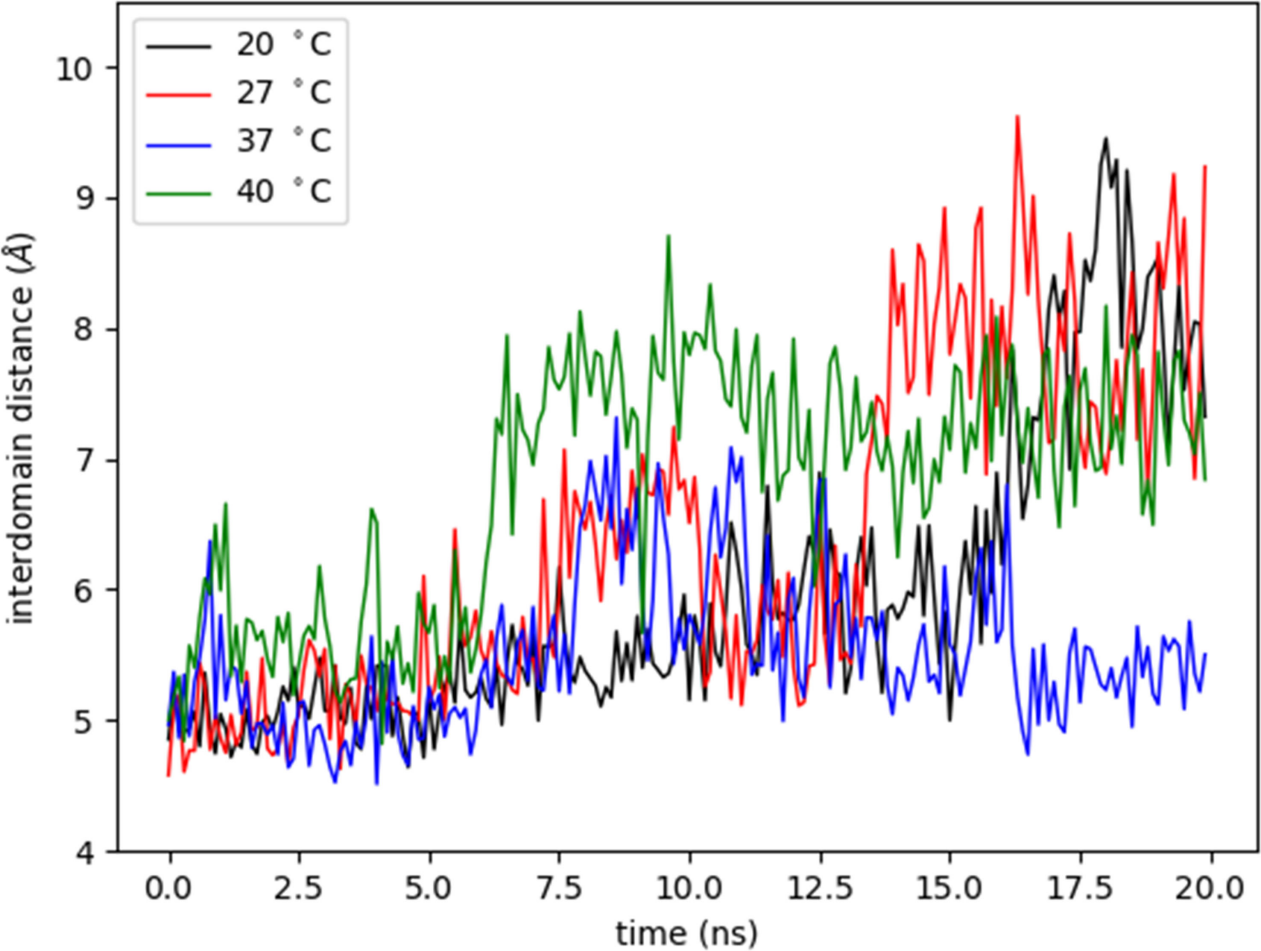
Variation of interdomain distance with time at different temperatures. The interdomain distance was measured as the distance (Å) between CA atom of residue 251 from β-propeller domain and CA atom of residue 619 from catalytic domain at different temperatures (°C) during 20 nanoseconds (ns).

The OPB enzyme model assessed in this work consists of two separate domains: a discontinuous catalytic domain containing an N-terminal region (residues 1–93) and a C-terminal region (residues 455-730); also, a seven-bladed β-propeller domain, with each blade composed of four β-strands (residues 103-446). The domains are linked together by a hinge region composed of two linear polypeptide strands (residues 94-102 and 447-454) (white regions in **Figure 7A**), showing that one possibility of substrate entry into OPB is the loosening of the interaction between the interdomain residues. This is related to the opening in the interdomain region caused by a hinge-like motion between them, which allows substrates to enter the cavity (Herndon et al., 1969; Kaushik and Sowdhamini, 2011). This motion is shown by the interdomain distance along the trajectories at different temperatures (**Supplementary Movie 1**), computed as the distance between residue 251 from β-propeller domain and residue 619 from catalytic domain (**Figure 8**). The hinge A (residues 94-102) connects the N-terminal segment of the catalytic domain with the first β-propeller β-strand. The hinge B (residues 447-454) connects the last β-propeller strand with the rest of the catalytic domain (C-terminal region). Hinge A remains unaltered at different temperatures, but, on the other hand, hinge B exhibits an amplification of the overall fluctuations as the temperature is increased. The progression of interdomain distance increased along the trajectories, more significantly at 20°C, 27°C and 40°C. At 37°C, the enzyme maintained the values of the interdomain distance, meaning the enzyme´ cavity did not open to expose the catalytic triad.

**Figure 9:**
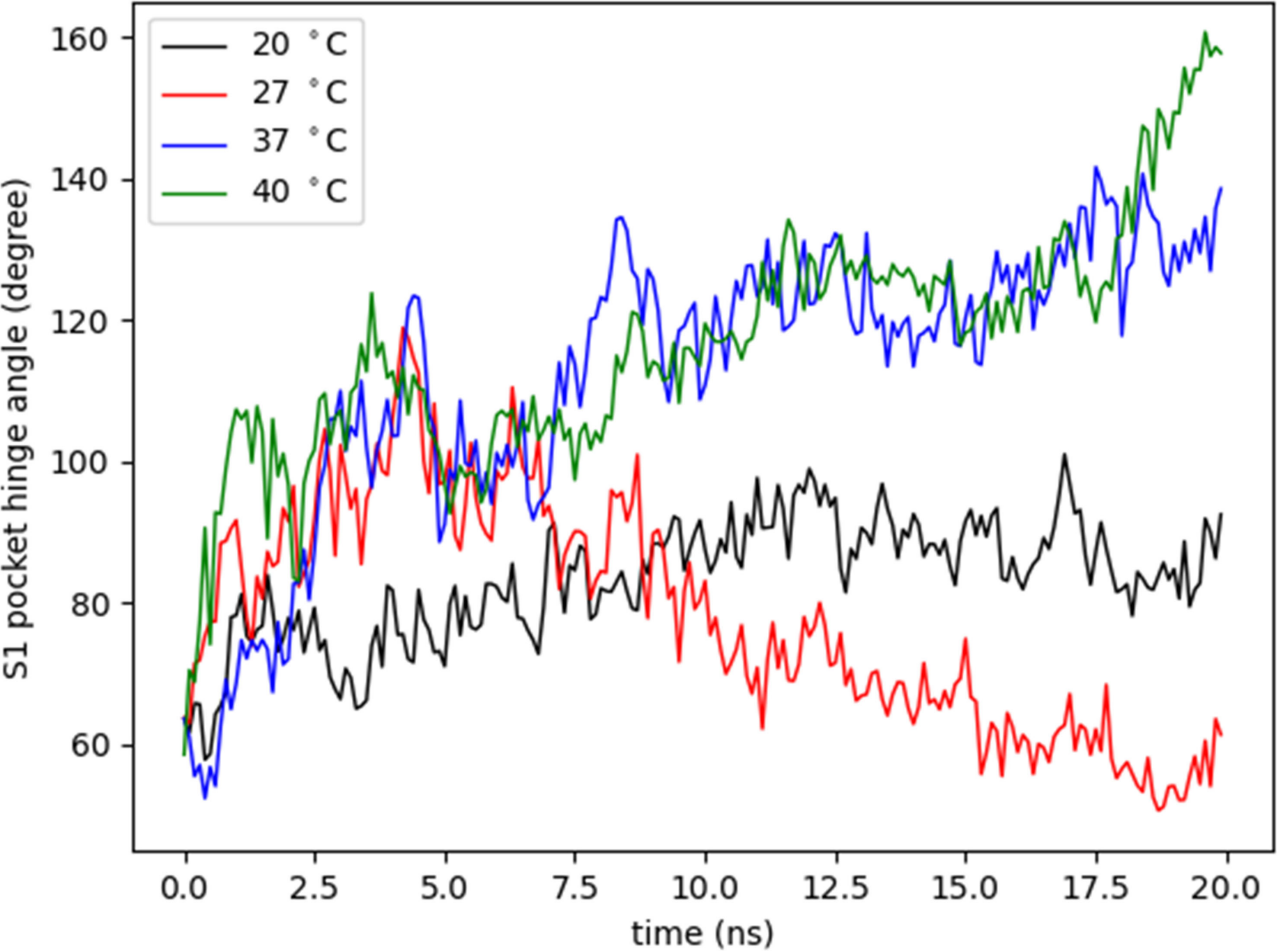
Variation of S1 pocket hinge angle. The S1 pocket hinge angle (degree) was measured as the angle between CA atoms of residues Arg389, Arg399, and Ser405 to describe the opening of region 5 at different temperatures (°C) during 20 nanoseconds (ns).

For this study, a model of the subtilisin-like catalytic domain (residues 85-470) was created, in which the catalytic triad consists of residues Asp97, His130, and Ser405 (dotted black lines in **Figure 7B**). At the subtilisin-like catalytic domain, six flexible regions situated among β-strands and α-helices showed the greatest fluctuations in RMSF (pale orange in **Figure 7B**). In the model: region 1 (residues 146-168) is between helix α1 and strand β2; region 2 (residues 210-230) between helix α2 and strand β5; regions 3 and 4 (residues 301-340 and residues 351-358, respectively) between β-strands β7 and β8; region 5 (residues 379-392) between β-strands β9 and β10; and region 6 (residues 424-453) between α-helices α4 and α5. Residues of the catalytic triad Asp97, His130, and Ser405 are in strand β1, helix α1, and helix α4, respectively. These residues exhibited RMSF values ~1Å, therefore, the catalytic triad displayed a smaller amplitude of fluctuations when compared to other residues of the catalytic domain. The angle between CA atoms of residues Arg389, Arg399, and Ser405 (S1 pocket hinge angle) described the “opening” of region 5. The motion of this region along the trajectory depicts the transition into an open conformation of the S1 pocket (**Supplementary Movie 2**). In **Figure 9**, the evolution in time of the S1 pocket hinge angle at different temperatures indicated a higher “opening” of region 5 during the simulations at 37°C and 40°C.

## Discussion

In South America, CL and ML are mainly caused by *L. (V.) braziliensis*, with a variable frequency according to epidemiological and clinical studies (Zerpa and Ponte-Sucre, 2013; Silva et al., 2017). *L. (V.) braziliensis* isolates from this region have been described as polyclonal populations with genetic and phenotypic diverse profiles (Cupolillo et al., 2003; Adaui et al., 2011b; Fernandes et al., 2016). Altogether, the weakness behind understanding this diverse scenario is the lack of factors that better characterize the phenotype variations within the same species. In the search for these factors, our group separated different phenotypes based on *in vitro* Sb^III^ susceptibility and serine proteases activity (Zabala-Peñafiel et al., 2021). In the present study, we continue in the search for possible discriminatory elements focusing on serine proteases, subtilisins and oligopeptidase B from *L. (V.) braziliensis*, and propose a relationship between them and the parasites’ *in vitro* fitness.

In fact, the clinical isolates, cultured under specific *in vitro* conditions, showed heterogeneous fitness profiles characterized as two groups based on promastigotes growth speed. Also, peritoneal macrophages infection showed small parasitophorous vacuoles as it is common in infections by *L. (V.) braziliensis* (Zauli-Nascimento et al., 2010; Fernandes et al., 2016). However, the infection by almost all isolates showed similar high infection rates, as previously observed with other *L. (V.) braziliensis* clinical isolates (Fernandes et al., 2016). These results led us to reflect that only the *in vitro* analyses, simulating conditions of both parasite life cycle microenvironments, are not sufficient to depict the phenotypic heterogeneity of these isolates. Therefore, we advanced in the phenotypic characterization based on the parasite impact within these microenvironments.

As a broader view of each clinical isolate and their *in vitro* impact under mammalian host conditions, the production of inflammatory mediators was assessed in macrophage supernatants. NO was better detected in the infection of some isolates which was not seen in another study (Fernandes et al., 2016). Meanwhile, the highest values of TNF-α (> 50 pg/mL) were secreted only in four infections. Additionally, high IL-6 (> 200 pg/mL) values were detected in infection by three isolates while this cytokine was not detected in other studies using *L. (V.) braziliensis* promastigotes to infect peritoneal macrophages (Fernandes et al., 2016) and peripheral blood mononuclear cells (Gomes et al., 2014). IL-6 production, a pleiotropic cytokine associated with Th2 differentiation and ML manifestations (Diehl and Rincón, 2002; Castellucci et al., 2012), reveals differential parasite fitness directly affecting host cell immune responses. Together, these results evidence the heterogeneity within the same species, which may indicate an adaptive advantage for these parasites.

In addition, the mentioned heterogeneity may also be related to these parasites’ capacity to synthesize proteins that influence their interaction with the hosts, resulting in their survival. In this context, it has been described that *L. (V.) braziliensis* clinical isolates have different gene expression profiles throughout their life cycle (Adaui et al., 2011a). This was also previously reported in the context of *L. (V.) braziliensis* clinical isolates heterogeneity based on higher expression of S13 gene in axenic amastigotes, and lowest expression of S28 gene in both promastigote and axenic amastigote forms (Zabala-Peñafiel et al., 2021). Moreover, advancing on the exploratory search and understanding of these heterogeneous phenotypes, based on serine proteases profiles, we describe an increased S13 and OPB expression in intracellular amastigotes of *L.* (*V.*) *braziliensis* clinical isolates, while S28 was not significantly expressed here and in our previous publication (Zabala-Peñafiel et al., 2021).

Furthermore, OPB gene expression was significantly higher in the intracellular amastigote than in the promastigote forms of *L. (V.) braziliensis* (Gamboa et al., 2007) and *L. (L.) donovani* (Swenerton et al., 2011), suggesting its potential as a stage biomarker. Moreover, *L. (L.) major* OPB knockouts promastigotes were able to infect macrophages but at a half rate than wild type promastigotes (Munday et al., 2011). Additionally, *L. (L.) donovani* OPB knockouts were able to infect macrophages but activated macrophages M1 phenotype with higher expression of TNF receptors and IFN-activated proteins (Swenerton et al., 2011). In addition, the correlation between serine proteases gene expression and inflammatory mediators was assessed here yielding a moderate positive Pearson correlation between S13 and OPB gene expression with IL-6 and TNF-α is an indicator that both innate immune response and pro-inflammatory cytokines are acting during the initial infection phase in line with the expression of these proteases. A fact strengthened with the negative correlation between S13 and NO production, and weak correlation between OPB and NO. These analyses add up to the evidence that S13 and OPB gene expression is more related with the parasite stage in the vertebrate host while this is not the case for S28 gene (**Supplementary Table 4**).

Moreover, temperature changes have an effect on molecular and morphological processes of *Leishmania* spp. adaptation and it is considered as an inducer factor of differential gene expression during differentiation (Zilberstein and Shapira, 1994). It is proposed that regulatory elements present in the 3’UTR sequences can act on gene expression of *Leishmania* spp. (Boucher et al., 2002; McNicoll et al., 2006; Leifso et al., 2007), as suggested for regulation of gp63 expression in *L. (L.) chagasi* (Ramamoorthy et al., 1995) and cysteine proteinase B in *L. (V.) braziliensis* (Gomes et al., 2017). Our data, concerning S13 and OPB 3’UTR sequences secondary structures, are predictive evidence that this region from S13 gene is more stable in mammalian host temperature, while this structure in OPB gene is able to tolerate temperature variation, both at sandfly vector and mammalian hosts temperatures. The presence of hairpin structures found in OPB and S13 3’UTR sequences suggests an active region of mRNA for both gene expression independent of the temperature used. 3´UTR are considered essential guiders of RNA folding during interaction with ribozyme, protection of mRNA to degradation, and recognition motif for RNA binding proteins (Roth et al., 1992; Bhasin et al., 1999; Svoboda and Cara, 2006). Interestingly, the prediction of a pseudoknot structure in the S13 3’UTR region at 29 to 36°C is an indication that this gene has a particular profile at these temperatures. Pseudoknot structures are related to a feedback regulation in gene expression by specific recognitions (Peselis and Serganov, 2014). This *in silico* data suggests that serine proteases gene expression is regulated by the temperature variations to which the parasites are exposed.

It is important to bear in mind that the mentioned changes are the parasite’ adaptive response to different environments, which are mainly governed by modulation of gene expression (Clayton, 2002). However, transcription mechanisms of *Leishmania* spp. are unusual since they regulate mRNA abundance through post-transcription mechanisms which, in result, does not directly correlate with protein amounts (Clayton, 2002; Campbell et al., 2003). The data regarding mRNA transcription showed that OPB and S13 have different gene expression patterns in both parasite forms and *in silico* assays of 3′UTR sequences suggest that these genes have a post-translational regulation under the distinct temperatures assessed. Nevertheless, future experiments are necessary to determine protein levels of these proteases in the different stages of the isolates.

Based on the differences of S13 and OPB expression in promastigotes and intracellular amastigotes, which reflects the genome plasticity experimented by the parasite throughout the life cycle, we advanced in other predictive analysis assessing the structural stability of both enzymes dependent on temperature variations.

The relative interdomain movements are suggested to be essential for the OPB enzyme action (Szeltner et al., 2004; Shan et al., 2005; Kaushik and Sowdhamini, 2011). The rise in temperature induced differences observed in the fluctuation of the two linear polypeptide strands of the hinge region. The enzyme opening is demonstrated to lead to the breakdown of the catalytic triad (Harmat et al., 2011), and the analysis of some OPB models showed that in open form there is a disconnection of the catalytic triad and the distortion of the contact interface between catalytic and β-propeller domains. This mechanism may explain the OPB catalytic efficiency in promastigotes and amastigotes microenvironments that represent different temperature substrate sources, as previously described for this enzyme due to substrate-dependent temperature sensitivity (Polgár, 1999).

Regarding the S13 enzyme structure, the rise in temperature also led to an overall increase of RMSF values. In general, for subtilisin homologues, at least four binding sites in the active groove are involved in the interaction with substrates (Withers-Martinez et al., 2002). In particular, the S1 pocket affects the dynamics of the substrate-binding region (Liu et al., 2010). Additionally, the fluctuations in regions 5 and 6 are related to the induction of an open conformation of the enzyme´ S1 pocket (Liu et al., 2010). The trajectory data of S1 pocket hinge angle shows that this pocket retained a longer open conformational state along the simulation at 37°C, which is the temperature the parasite experiences within the mammalian host cells. Proteins´ flexibility, in the context of adaptation to temperature, has a central role to link structure and function (Dong et al., 2018). The largest amount of structural fluctuation commonly lies in regions involved in the catalytic activity. This might already be sufficient to induce affinity-regulation, conformational change and it can be explained by the fact that it is easier for substrates to bind to the S1 pocket in an open conformational state. This predictive evidence, about the behavior of the tridimensional structure of the S13 enzyme, indicates that the enzyme has a better catalytic action in the amastigote forms which also have higher expression of S13 gene.

Proteases are virulence factors that act in the manipulation of the immune system and the development of infection in the hosts (McKerrow et al., 2006; Silva-Almeida et al., 2012). The *in vitro* assays carried out here bring together elements that suggest the importance of the expression of the S13 and OPB genes predominantly in intracellular amastigotes. While *in silico* assays points to the mRNA and enzymes, encoded by these genes, that have a structural potential to act in different temperatures. Together, we highlight the complex dynamics of gene expression and structural stability of serine proteases indicating a possible regulation due to plasticity of its biologic cycle guided by temperature changes.

## Data Availability Statement

The original contributions presented in the study are included in the article/**Supplementary Material**, further inquiries can be directed to the corresponding authors.

## Author Contributions

AZ-P conducted the *in vitro* experiments, data analysis, and wrote the original manuscript. FS-S and AAMLB conducted the *in silico* experiments. CRA, GD-L, LC-F, and FS-S helped in the experiments planning and execution. FC-S, AF, and LM provided the frozen parasite isolates and information about the clinical origin of the isolates. GD-L, FC-S, and CRA critically reviewed the data and final manuscript. All authors contributed to the article and approved the submitted version.

## Funding

This study was financed in part by the Conselho Nacional de Desenvolvimento Científico e Tecnológico - Brasil (CNPq: 301744/2019-0), Fundação de Amparo à Pesquisa do Estado do Rio de Janeiro - Brasil (FAPERJ: E-26/200.799/2021; E-26/202.661/2021; E-26/010.002021/2019; E-26/204.188/2021) and the Coordenação de Aperfeiçoamento de Pessoal de Nível Superior - Brasil (CAPES: Finance Code 001).

## Conflict of Interest

The authors declare that the research was conducted in the absence of any commercial or financial relationships that could be construed as a potential conflict of interest.

## Publisher’s Note

All claims expressed in this article are solely those of the authors and do not necessarily represent those of their affiliated organizations, or those of the publisher, the editors and the reviewers. Any product that may be evaluated in this article, or claim that may be made by its manufacturer, is not guaranteed or endorsed by the publisher.

## Supplementary Material

Supplementary Movie 1 Molecular Dynamics simulations of the OPB at 27°C (left view) and 37°C (right view). The entire enzyme is represented in NewCartoon (blue) and QuickSurf (white). CA atoms of residues Leu251 and Thr619 are represented in VDW (red) linked by a dashed line representing the distance between these two atoms (black). The residues of the catalytic triad, Ser577, His697, and Asp662, are represented in VDW (red). The progressions of the distance between CA atoms of residues Leu251 and Thr619 exhibited a similar behavior for the initial steps of the simulations but showed larger distance at the end of the trajectories (left view).

Supplementary Movie 2 Molecular Dynamics simulations of the Subtilisin 13 catalytic domain at 27°C (left view) and 37°C (right view). The domain is represented in NewCartoon (blue) and QuickSurf (white). The region 5 in the Subtilisin catalytic domain (residues 379-392) is represented in NewCartoon and QuickSurf, both in green. The residues of the catalytic triad, Asp97, His130, and Ser405, are represented in VDW (red). CA atoms of residues Arg389, Arg399, and Ser405 are represented in VDW (red). The angle formed by these three atoms (black) describes the “opening” of the region 5. The motion of the region 5 along the trajectory depicts the transition to an open conformation of S1 pocket at 37°C (right view).

## References

[B1] Adaui V. Castillo D. Zimic M. Gutierrez A. Decuypere S. Vanaerschot M.. (2011a). Comparative Gene Expression Analysis Throughout the Life Cycle of Leishmania Braziliensis: Diversity of Expression Profiles Among Clinical Isolates. PloS Neglect. Trop. Dis. 5 (5), e1021. doi: 10.1371/journal.pntd.0001021 PMC3091834 21572980

[B2] Adaui V. Schnorbusch K. Zimic M. Gutiérrez A. Decuypere S. Vanaerschot M.. (2011b). Comparison of Gene Expression Patterns Among Leishmania Braziliensis Clinical Isolates Showing a Different *In Vitro* Susceptibility to Pentavalent Antimony. Parasitology 138 (2), 183–193. doi: 10.1017/S0031182010001095 20678296

[B3] Akhoundi M. Kuhls K. Cannet A. Votýpka J. Marty P. Delaunay P.. (2016). A Historical Overview of the Classification, Evolution, and Dispersion of Leishmania Parasites and Sandflies. PloS Neglect. Trop. Dis. 10 (3), e0004349. doi: 10.1371/journal.pntd.0004349 PMC4777430 26937644

[B4] Alcolea P. J. Alonso A. García-Tabares F. Mena M. C. Ciordia S. Larraga V. (2016). Increased Abundance of Proteins Involved in Resistance to Oxidative and Nitrosative Stress at the Last Stages of Growth and Development of Leishmania Amazonensis Promastigotes Revealed by Proteome Analysis. PloS One 11 (10), e0164344. doi: 10.1371/journal.pone.0164344 27776144 PMC5077082

[B5] Alcolea P. J. Alonso A. Gómez M. J. Sánchez-Gorostiaga A. Moreno-Paz M. González-Pastor E.. (2010). Temperature Increase Prevails Over Acidification in Gene Expression Modulation of Amastigote Differentiation in Leishmania Infantum. BMC Genomics 11 (1), 31. doi: 10.1186/1471-2164-11-31 20074347 PMC2845110

[B6] Alvar J. Vélez I. Bern C. Herrero M. Desjeux P. Cano J.. (2012). Leishmaniasis Worldwide and Global Estimates of Its Incidence. PloS One 7 (5), e35671. doi: 10.1371/journal.pone.0035671 22693548 PMC3365071

[B7] Alves C. Santos-de-Souza R. dos Santos Charret K. Côrtes L. M. C. Sá-Silva M. Barral-Veloso L.. (2018). Understanding Serine Proteases Implications on Leishmania Spp Lifecycle. Exp. Parasitol. 184, 67–81. doi: 10.1016/j.exppara.2017.11.008 29175018

[B8] Bates P. A. (2007). Transmission of Leishmania Metacyclic Promastigotes by Phlebotomine Sand Flies. Int. J. Parasitol. 37 (10–3), 1097–1106. doi: 10.1016/j.ijpara.2007.04.003 17517415 PMC2675784

[B9] Bhasin A. Goryshin I. Y. Reznikoff W. S. (1999). Hairpin Formation in Tn5 Transposition. J. Biol. Chem. 274 (52), 37021–37029. doi: 10.1074/jbc.274.52.37021 10601258

[B10] Boucher N. Wu Y. Dumas C. Dubé M. Sereno D. Breton M.. (2002). A Common Mechanism of Stage-Regulated Gene Expression in Leishmania Mediated by a Conserved 3′-Untranslated Region Element*. J. Biol. Chem. 277 (22), 19511–19520. doi: 10.1074/jbc.M200500200 11912202

[B11] Campbell D. A. Thomas S. Sturm N. R. (2003). Transcription in Kinetoplastid Protozoa: Why be Normal? Microbes Infect. 5 (13), 1231–1240. doi: 10.1016/j.micinf.2003.09.005 14623019

[B12] Castellucci L. Jamieson S. E. Almeida L. Oliveira J. Guimarães L. H. Lessa M.. (2012). Wound Healing Genes and Susceptibility to Cutaneous Leishmaniasis in Brazil. Infect. Genet. Evol. 12 (5), 1102–1110. doi: 10.1016/j.meegid.2012.03.017 22554650 PMC3372530

[B13] Clayton C. E. (2002). Life Without Transcriptional Control? From Fly to Man and Back Again. EMBO J. 21 (8), 1881–1888. doi: 10.1093/emboj/21.8.1881 11953307 PMC125970

[B14] Cupolillo E. Brahim L. R. Toaldo C. B. de Oliveira-Neto M. P. de Brito M. E. F. Falqueto A.. (2003). Genetic Polymorphism and Molecular Epidemiology of Leishmania (Viannia) Braziliensis From Different Hosts and Geographic Areas in Brazil. J. Clin. Microbiol. 41 (7), 3126–3132. doi: 10.1128/JCM.41.7.3126-3132.2003 12843052 PMC165365

[B15] Cupolillo E. Grimaldi G. Momen H. (1994). A General Classification of New World Leishmania Using Numerical Zymotaxonomy. Am. J. Trop. Med. Hyg. 50 (3), 296–311. doi: 10.4269/ajtmh.1994.50.296 8147488

[B16] Cysne-Finkelstein L. Silva-Almeida M. Pereira B. dos Santos Charret K. Bertho Á.L. Bastos L.. (2018). Evidence of Subpopulations With Distinct Biological Features Within a Leishmania (Viannia) Braziliensis Strain. Protist 169 (1), 107–121. doi: 10.1016/j.protis.2017.11.004 29482071

[B17] Depledge D. P. Evans K. J. Ivens A. C. Aziz N. Maroof A. Kaye P. M. Smith D. F.. (2009). Comparative Expression Profiling of Leishmania: Modulation in Gene Expression Between Species and in Different Host Genetic Backgrounds. PloS Neglect. Trop. Dis. 3 (7), e476. doi: 10.1371/journal.pntd.0000476 PMC2701600 19582145

[B18] Diehl S. Rincón M. (2002). The Two Faces of IL-6 on Th1/Th2 Differentiation. Mol. Immunol. 39 (9), 531–536. doi: 10.1016/s0161-5890(02)00210-9 12431386

[B19] Dong Y. Liao M. Meng X. Somero G. N. (2018). Structural Flexibility and Protein Adaptation to Temperature: Molecular Dynamics Analysis of Malate Dehydrogenases of Marine Molluscs. Proc. Natl. Acad. Sci. 115 (6), 1274–1279. doi: 10.1073/pnas.1718910115 29358381 PMC5819447

[B20] Dostálová A. Volf P. (2012). Leishmania Development in Sand Flies: Parasite-Vector Interactions Overview. Parasit. Vectors 5 (1), 276. doi: 10.1186/1756-3305-5-276 23206339 PMC3533922

[B21] Ennes-Vidal V. Vitório B. Menna-Barreto R. Pitaluga A. Gonçalves-da-Silva S. A. Branquinha M. H.. (2019). Calpains of Leishmania Braziliensis: Genome Analysis, Differential Expression, and Functional Analysis. Mem. Do Inst. Oswaldo Cruz 114, e190147. doi: 10.1590/0074-02760190147 PMC6759280 31553371

[B22] Fernandes A. C. B. S. Pedroso R. B. de Mello T. F. P. Donatti L. Venazzi E. A. S. Demarchi I. G.. (2016). *In Vitro* Characterization of Leishmania (Viannia) Braziliensis Isolates From Patients With Different Responses to Glucantime® Treatment From Northwest Paraná, Brazil. Exp. Parasitol. 167, 83–93. doi: 10.1016/j.exppara.2016.05.003 27181585

[B23] Fiebig M. Kelly S. Gluenz E. (2015). Comparative Life Cycle Transcriptomics Revises Leishmania Mexicana Genome Annotation and Links a Chromosome Duplication With Parasitism of Vertebrates. PloS Pathog. 11 (10), e1005186. doi: 10.1371/journal.ppat.1005186 26452044 PMC4599935

[B24] Figueiredo de Sá B. S. L. Rezende A. Melo Neto O. Brito M. E. Brandão Filho S. (2019). Identification of Divergent Leishmania (Viannia) Braziliensis Ecotypes Derived From a Geographically Restricted Area Through Whole Genome Analysis. PloS Neglect. Trop. Dis. 13 (6), e0007382. doi: 10.1371/journal.pntd.0007382 PMC6581274 31170148

[B25] Folgueira C. Quijada L. Soto M. Abanades D. R. Alonso C. Requena J. M. (2005). The Translational Efficiencies of the Two Leishmania Infantum HSP70 mRNAs, Differing in Their 3′-Untranslated Regions, Are Affected by Shifts in the Temperature of Growth Through Different Mechanisms *. J. Biol. Chem. 280 (42), 35172–35183. doi: 10.1074/jbc.M505559200 16105831

[B26] Gamboa D. Van Eys G. Victoir K. Torres K. Adaui V. Arevalo J.. (2007). Putative Markers of Infective Life Stages in *Leishmania (Viannia) Braziliensis* . Parasitology 134 (12), 1689–1698. doi: 10.1017/S003118200700306X 17897481

[B27] Gomes C. M. Ávila L. R. Pinto S. A. Duarte F. B. Pereira L. I. A. Abrahamsohn I. A.. (2014). Leishmania Braziliensis Amastigotes Stimulate Production of IL-1β, IL-6, IL-10 and TGF-β by Peripheral Blood Mononuclear Cells From Nonendemic Area Healthy Residents. Parasit. Immunol. 36 (5), 225–231. doi: 10.1111/pim.12109 24575815

[B28] Gomes C. B. Silva F. S. Charret K. D. S. Pereira B. A. S. Finkelstein L. C. Santos-de-Souza R.. (2017). Increasing in Cysteine Proteinase B Expression and Enzymatic Activity During *In Vitro* Differentiation of Leishmania (Viannia) Braziliensis: First Evidence of Modulation During Morphological Transition. Biochimie 133, 28–36. doi: 10.1016/j.biochi.2016.11.015 27919786

[B29] Gossage S. M. Rogers M. E. Bates P. A. (2003). Two Separate Growth Phases During the Development of Leishmania in Sand Flies: Implications for Understanding the Life Cycle. Int. J. Parasitol. 33 (10), 1027–1034. doi: 10.1016/S0020-7519(03)00142-5 13129524 PMC2839921

[B30] Harmat V. Domokos K. Menyhárd D. K. Palló A. Szeltner Z. Szamosi I.. (2011). Structure and Catalysis of Acylaminoacyl Peptidase. J. Biol. Chem. 286 (3), 1987–1998. doi: 10.1074/jbc.M110.169862 21084296 PMC3023495

[B31] Herndon J. H. Steinberg D. Uhlendorf B. W. Fales H. M. (1969). Refsum’s Disease: Characterization of the Enzyme Defect in Cell Culture. J. Clin. Invest. 48 (6), 1017–1032. doi: 10.1172/JCI106058 4181593 PMC322316

[B32] Holzer T. R. McMaster W. R. Forney J. D. (2006). Expression Profiling by Whole-Genome Interspecies Microarray Hybridization Reveals Differential Gene Expression in Procyclic Promastigotes, Lesion-Derived Amastigotes, and Axenic Amastigotes in Leishmania Mexicana. Mol. Biochem. Parasitol. 146 (2), 198–218. doi: 10.1016/j.molbiopara.2005.12.009 16430978

[B33] Humphrey W. Dalke A. Schulten K. (1996). VMD: Visual Molecular Dynamics. J. Mol. Graphics 14 (1), 33–38. doi: 10.1016/0263-7855(96)00018-5 8744570

[B34] Jorgensen W. L. Chandrasekhar J. Madura J. D. Impey R. W. Klein M. L. (1998). Comparison of Simple Potential Functions for Simulating Liquid Water. J. Chem. Phys. 79 (2), 926. doi: 10.1063/1.445869

[B35] Kaushik S. Sowdhamini R. (2011). Structural Analysis of Prolyl Oligopeptidases Using Molecular Docking and Dynamics: Insights Into Conformational Changes and Ligand Binding. PloS One 6 (11), e26251. doi: 10.1371/journal.pone.0026251 22132071 PMC3223163

[B36] Leifso K. Cohen-Freue G. Dogra N. Murray A. McMaster W. R. (2007). Genomic and Proteomic Expression Analysis of Leishmania Promastigote and Amastigote Life Stages: The Leishmania Genome is Constitutively Expressed. Mol. Biochem. Parasitol. 152 (1), 35–46. doi: 10.1016/j.molbiopara.2006.11.009 17188763

[B37] Liu S.-Q. Meng Z.-H. Fu Y.-X. Zhang K.-Q. (2010). Insights Derived From Molecular Dynamics Simulation Into the Molecular Motions of Serine Protease Proteinase K. J. Mol. Modeling 16 (1), 17–28. doi: 10.1007/s00894-009-0518-x 19466463

[B38] Livak K. J. Schmittgen T. D. (2001). Analysis of Relative Gene Expression Data Using Real-Time Quantitative PCR and the 2–ΔΔct Method. Methods 25 (4), 402–408. doi: 10.1006/meth.2001.1262 11846609

[B39] MacKerell J. Bashford D. Bellott M. Dunbrack J. Evanseck J. D. Field M. J.. (1998). All-atom Empirical Potential for Molecular Modeling and Dynamics Studies of Proteins. J. Phys. Chem. B. 102 (18), 3586–3616. doi: 10.1021/jp973084f 24889800

[B40] Marco J. D. Barroso P. A. Locatelli F. M. Cajal S. Hoyos C. L. Nevot M.. (2015). Multilocus Sequence Typing Approach for a Broader Range of Species of Leishmania Genus: Describing Parasite Diversity in Argentina. Infect. Genet. Evol. 30, 308–317. doi: 10.1016/j.meegid.2014.12.031 25558029

[B41] Marlow M. Boité M. Ferreira G. Steindel M. Cupolillo E. (2014). Multilocus Sequence Analysis for Leishmania Braziliensis Outbreak Investigation. PloS Neglect. Trop. Dis. 8 (2), e2695. doi: 10.1371/journal.pntd.0002695 PMC3923721 24551258

[B42] Matlashewski G. (2001). Leishmania Infection and Virulence. Med. Microbiol. Immunol. 190 (1–2), 37–42. doi: 10.1007/s004300100076 11770107

[B43] McKerrow J. H. Caffrey C. Kelly B. Loke P. Sajid M. (2006). PROTEASE IN PARASITIC DISEASES. Annu. Rev. Pathol.: Mech. Dis. 1 (1), 497–536. doi: 10.1146/annurev.pathol.1.110304.100151 18039124

[B44] McNicoll F. Drummelsmith J. Müller M. Madore E. Boilard N. Ouellette M.. (2006). A Combined Proteomic and Transcriptomic Approach to the Study of Stage Differentiation in Leishmania Infantum. Proteomics 6 (12), 3567–3581. doi: 10.1002/pmic.200500853 16705753

[B45] Munday J. C. McLuskey K. Brown E. Coombs G. H. Mottram J. C. (2011). Oligopeptidase B Deficient Mutants of Leishmania Major. Mol. Biochem. Parasitol. 175 (1), 49–57. doi: 10.1016/j.molbiopara.2010.09.003 20883728 PMC3130898

[B46] Patino L. H. Muñoz M. Cruz-Saavedra L. Muskus C. Ramírez J. D. (2020). Genomic Diversification, Structural Plasticity, and Hybridization in Leishmania (Viannia) Braziliensis. Front. Cell. Infect. Microbiol. 10. doi: 10.3389/fcimb.2020.582192 PMC7596589 33178631

[B47] Peselis A. Serganov A. (2014). Structure and Function of Pseudoknots Involved in Gene Expression Control. Wiley Interdiscip. Rev. RNA 5 (6), 803–822. doi: 10.1002/wrna.1247 25044223 PMC4664075

[B48] Phillips J. C. Braun R. Wang W. Gumbart J. Tajkhorshid E. Villa E.. (2005). Scalable Molecular Dynamics With NAMD. J. Comput. Chem. 26 (16), 1781–1802. doi: 10.1002/jcc.20289 16222654 PMC2486339

[B49] Polgár L. (1999). Oligopeptidase B: A New Type of Serine Peptidase With a Unique Substrate-Dependent Temperature Sensitivity. Biochemistry 38 (47), 15548–15555. doi: 10.1021/bi991767a 10569938

[B50] Quaresma P. F. de Brito C. F. A. Rugani J. M. N. de Moura Freire J. de Paula Baptista R. Moreno E. C.. (2018). Distinct Genetic Profiles of Leishmania (Viannia) Braziliensis Associate With Clinical Variations in Cutaneous-Leishmaniasis Patients From an Endemic Area in Brazil. Parasitology 145 (9), 1161–1169. doi: 10.1017/S0031182018000276 29526166

[B51] Queiroz A. Sousa R. Heine C. Cardoso M. Guimarães L. H. Machado P. R. L.. (2012). Association Between an Emerging Disseminated Form of Leishmaniasis and Leishmania (Viannia) Braziliensis Strain Polymorphisms. J. Clin. Microbiol. 50 (12), 4028–4034. doi: 10.1128/JCM.02064-12 23035200 PMC3503016

[B52] Ramamoorthy R. Swihart K. G. McCoy J. J. Wilson M. E. Donelson J. E. (1995). Intergenic Regions Between Tandem Gp63 Genes Influence the Differential Expression of Gp63 RNAs in Leishmania Chagasi Promastigotes *. J. Biol. Chem. 270 (20), 12133–12139. doi: 10.1074/jbc.270.20.12133 7744862

[B53] Rêgo F. D. da Rocha Lima A. C. V. M. Pereira A. A. S. Quaresma P. F. Pascoal-Xavier M. A. Shaw J. J.. (2018). Genetic Variant Strains of Leishmania (Viannia) Braziliensis Exhibit Distinct Biological Behaviors. Parasitol. Res. 117 (10), 3157–3168. doi: 10.1007/s00436-018-6014-4 30022292

[B54] Rochette A. Raymond F. Corbeil J. Ouellette M. Papadopoulou B. (2009). Whole-Genome Comparative RNA Expression Profiling of Axenic and Intracellular Amastigote Forms of Leishmania Infantum. Mol. Biochem. Parasitol. 165 (1), 32–47. doi: 10.1016/j.molbiopara.2008.12.012 19393160

[B55] Roth D. B. Menetski J. P. Nakajima P. B. Bosma M. J. Gellert M. (1992). V(D)J Recombination: Broken DNA Molecules With Covalently Sealed (Hairpin) Coding Ends in Scid Mouse Thymocytes. Cell 70 (6), 983–991. doi: 10.1016/0092-8674(92)90248-B 1356077

[B56] Séguin O. Descoteaux A. (2016). Leishmania, the Phagosome, and Host Responses: The Journey of a Parasite. Cell. Immunol. 309, 1–6. doi: 10.1016/j.cellimm.2016.08.004 27531526

[B57] Shan L. Mathews I. I. Khosla C. (2005). Structural and Mechanistic Analysis of Two Prolyl Endopeptidases: Role of Interdomain Dynamics in Catalysis and Specificity. Proc. Natl. Acad. Sci. 102 (10), 3599–3604. doi: 10.1073/pnas.0408286102 15738423 PMC553306

[B58] Silva-Almeida M. Carvalho L. O. Abreu-Silva A. L. Souza C. S. Hardoim D. J. Calabrese K. S. (2012). Extracellular Matrix Alterations in Experimental Leishmania Amazonensis Infection in Susceptible and Resistant Mice. Vet. Res. 43 (1), 10. doi: 10.1186/1297-9716-43-10 22316002 PMC3395857

[B59] Silva-Almeida M. Souza-Silva F. Pereira B. Ribeiro-Guimarães M. Alves C. R. (2014). Overview of the Organization of Protease Genes in the Genome of Leishmania Spp. Parasit. Vectors 7, 387. doi: 10.1186/1756-3305-7-387 25142315 PMC4158035

[B60] Silva J. Queiroz A. Moura I. Sousa R. S. Guimarães L. H. Machado P. R. L.. (2017). Dynamics of American Tegumentary Leishmaniasis in a Highly Endemic Region for Leishmania (Viannia) Braziliensis Infection in Northeast Brazil. PloS Neglect. Trop. Dis. 11 (11), e0006015. doi: 10.1371/journal.pntd.0006015 PMC5685640 29095818

[B61] Svoboda P. Cara A. (2006). Hairpin RNA: A Secondary Structure of Primary Importance. Cell. Mol. Life Sci. CMLS 63 (7), 901–908. doi: 10.1007/s00018-005-5558-5 16568238 PMC11136179

[B62] Swenerton R. K. Knudsen G. M. Sajid M. Kelly B. L. McKerrow J. H. (2010). Leishmania Subtilisin Is a Maturase for the Trypanothione Reductase System and Contributes to Disease Pathology. J. Biol. Chem. 285 (41), 31120–31129. doi: 10.1074/jbc.M110.114462 20675366 PMC2951185

[B63] Swenerton R. K. Zhang S. Sajid M. Medzihradszky K. F. Craik C. S. Kelly B. L.. (2011). The Oligopeptidase B of Leishmania Regulates Parasite Enolase and Immune Evasion. J. Biol. Chem. 286 (1), 429–440. doi: 10.1074/jbc.M110.138313 20961853 PMC3013002

[B64] Szeltner Z. Rea D. Juhász T. Renner V. Fülöp V. Polgár L. (2004). Concerted Structural Changes in the Peptidase and the Propeller Domains of Prolyl Oligopeptidase are Required for Substrate Binding. J. Mol. Biol. 340 (3), 627–637. doi: 10.1016/j.jmb.2004.05.011 15210359

[B65] Veloso L. B. de Oliveira Cardoso F. dos Santos Charret K. de Sá Silva M. P. de Castro Côrtes L. M. da Silva Calabrese K.. (2020). Detection of Metalloproteases and Cysteine Proteases RNA Transcripts of *Leishmania (Leishmania) Infantum* in Ear Edge Skin of Naturally Infected Dogs. BioMed. Res. Int. 2020, 1–8. doi: 10.1155/2020/2615787 PMC7333044 32685457

[B66] Withers-Martinez C. Saldanha J. W. Ely B. Hackett F. O’Connor T. Blackman M. J. (2002). Expression of Recombinant Plasmodium Falciparumsubtilisin-Like Protease-1 in Insect Cells: CHARACTERIZATION, COMPARISON WITH THE PARASITE PROTEASE, AND HOMOLOGY MODELING *. J. Biol. Chem. 277 (33), 29698–29709. doi: 10.1074/jbc.M203088200 12052828

[B67] World Health Organization (WHO) (2002). Urbanization: An Increasing Risk Factor for Leishmaniasis. Wkly. Epidemiol. Rec. 77 (44), 365–372. 12428426

[B68] Zabala-Peñafiel A. Dias-Lopes G. Cysne-Finkelstein L. Conceição-Silva F. Miranda L. de F. C.. (2021). Serine Proteases Profiles of Leishmania (Viannia) Braziliensis Clinical Isolates With Distinct Susceptibilities to Antimony. Sci. Rep. 11 (1), 14234. doi: 10.1038/s41598-021-93665-z 34244581 PMC8271011

[B69] Zauli-Nascimento R. C. Miguel D. C. Yokoyama-Yasunaka J. K. U. Pereira L. I. A. Pelli de Oliveira M. A. Ribeiro-Dias F.. (2010). *In Vitro* Sensitivity of Leishmania (Viannia) Braziliensis and Leishmania (Leishmania) Amazonensis Brazilian Isolates to Meglumine Antimoniate and Amphotericin B. Trop. Med. Int. Health: TM IH 15 (1), 68–76. doi: 10.1111/j.1365-3156.2009.02414.x 19874570

[B70] Zerpa O. Ponte-Sucre A. (2013). American Tegumentary Leishmaniasis. In: Ponte-Sucre, A., Diaz, E., Padrón-Nieves, M. (eds) Drug Resistance in Leishmania Parasites. (Vienna: Springer). 199–211. doi: 10.1007/978-3-7091-1125-3_10

[B71] Zilberstein D. Shapira M. (1994). The Role of pH and Temperature in the Development of Leishmania Parasites. Annu. Rev. Microbiol. 48 (1), 449–470. doi: 10.1146/annurev.mi.48.100194.002313 7826014

