# Supplementary material for: Novel Insights Into Leishmania (Viannia) braziliensis In Vitro Fitness Guided by Temperature Changes Along With Its Subtilisins and Oligopeptidase B
Source: Front Cell Infect Microbiol. 2022 Apr 21;12:805106. doi: 10.3389/fcimb.2022.805106 (PMC9069558; doi:10.3389/fcimb.2022.805106)
Supplement: Supplementary file 3 [file Image_1.pdf]

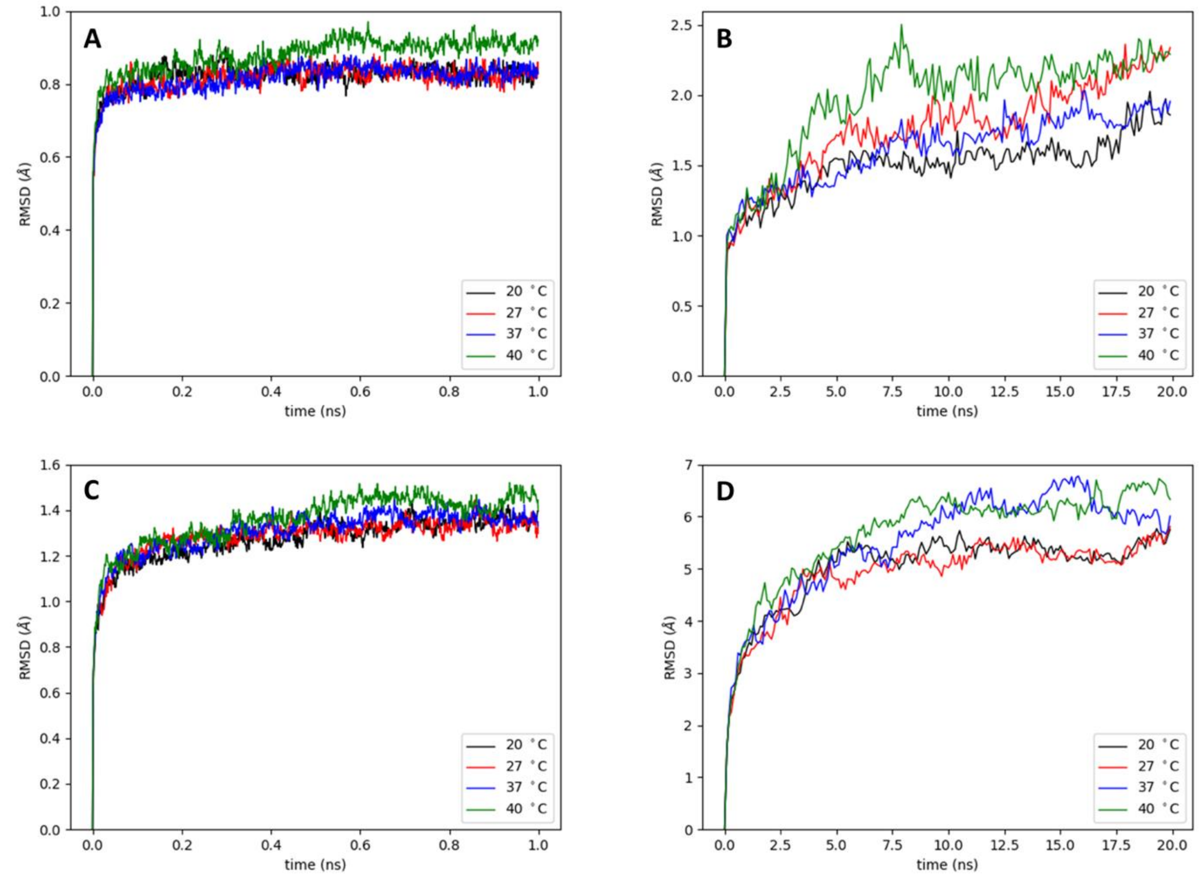

Supplementary figure 1. Root Mean Square Deviation values (RMSD) were assessed to show both protein structures equilibration for OPB and S13 enzymes before the production simulations (boxes A and C, respectively). Boxes B and D show RMSD values calculated along the production simulations, for OPB and S13, respectively. Both curves indicate the stabilization of the enzymes at different temperatures.
