# Supplementary material for: Novel Insights Into Leishmania (Viannia) braziliensis In Vitro Fitness Guided by Temperature Changes Along With Its Subtilisins and Oligopeptidase B
Source: Front Cell Infect Microbiol. 2022 Apr 21;12:805106. doi: 10.3389/fcimb.2022.805106 (PMC9069558; doi:10.3389/fcimb.2022.805106)
Supplement: Supplementary file 4 [file Image_2.pdf]

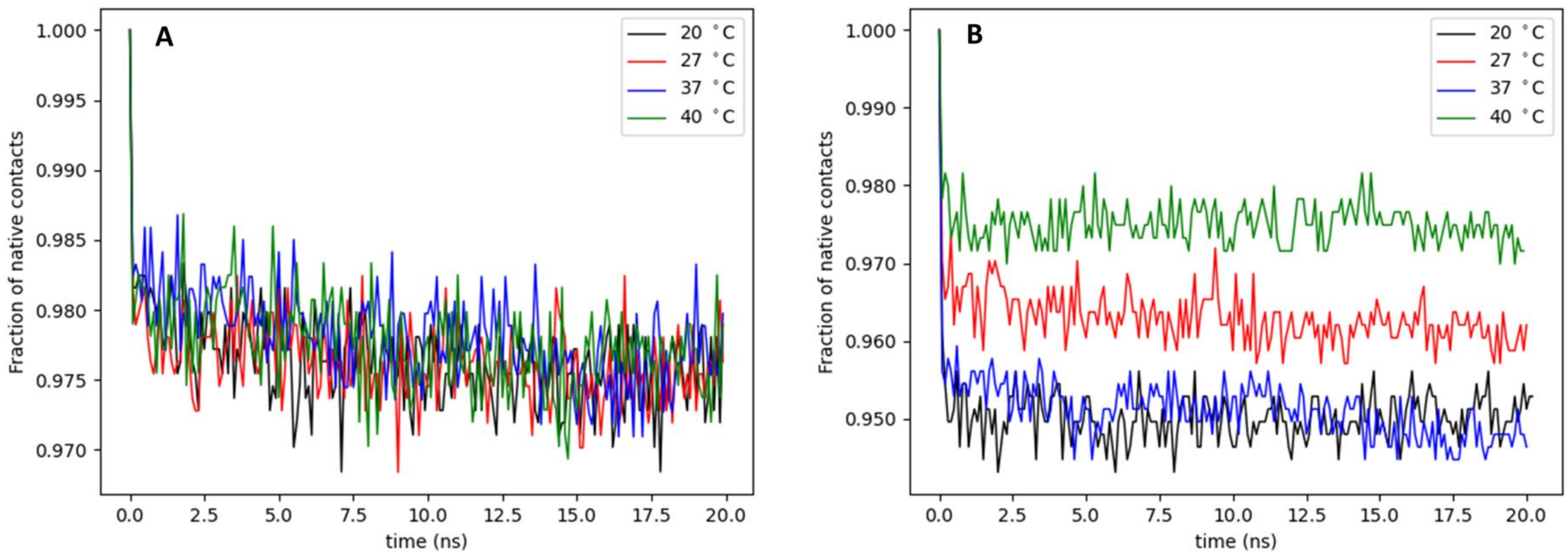


**Supplementary figure 2.** Native contacts. The enzymes´ intermolecular interaction, which occurred at the beginning of the simulations as an additional indicator of the enzyme stability. Native contacts curves for OPB (box A) and S13 (box B) show the enzymes maintained a fraction of initial contacts higher than 95% for both enzymes. These behaviors indicate that the complexes are not unfolding and reflect their stability at different temperatures.
