# Supplementary material for: Novel Insights Into Leishmania (Viannia) braziliensis In Vitro Fitness Guided by Temperature Changes Along With Its Subtilisins and Oligopeptidase B
Source: Front Cell Infect Microbiol. 2022 Apr 21;12:805106. doi: 10.3389/fcimb.2022.805106 (PMC9069558; doi:10.3389/fcimb.2022.805106)
Supplement: Supplementary file 5 [file Table_1.docx]

# Supplementary Table 1. Experimental information of *L. (V.) braziliensis* isolates used in this study

| ** Label* | *^ß^ Clinical Label* | *^+^Clusters* | *Promastigotes* | | | | |
| --- | --- | --- | --- | --- | --- | --- | --- |
|  |  |  | ***IC_50_ [mg/mL]* | | ****Protease residual activity [μmol min^-1^.mg of protein^-1^]* | | |
|  |  |  | *Sb^V^* | *Sb^III^* | *PMSF* | *AEBSF* | *TLCK* |
| 1 | R1 | Cluster 2 | 0.005 ± 0.27 | 0.008 ± 0.0008 | 644 ± 1.2 | 133 ± 0.8 | 14 ± 0.05 |
| 2 | NR1 | Cluster 5 | 0.908 ± 0.04 | 0.014 ± 0.0007 | 890 ± 0.05 | 60 ± 0.09 | 9 ± 0.03 |
| 3 | NR2 | Cluster 4 | 3.996 ± 1.4 | 0.008 ± 0.0003 | 576 ± 1.2 | 64 ± 0.08 | 9 ± 0.02 |
| 4 | R2 | Cluster 4 | 0.821 ± 0.09 | 0.003 ± 0.0002 | 548 ± 1.7 | 123 ± 0.05 | 44 ± 0.1 |
| 5 | R3 | Cluster 2 | 0.951 ± 0.2 | 0.015 ± 0.0007 | 554 ± 1.6 | 128 ± 0.06 | 35 ± 0.09 |
| 6 | R4 | Cluster 4 | 0.591 ± 0.07 | 0.007 ± 0.0004 | 592 ± 0.7 | 140 ± 0.03 | 14 ± 0.29 |
| 7 | NR3 | Cluster 3 | 0.449 ± 0.05 | 0.010 ± 0.0003 | 570 ± 0.8 | 457 ± 0.9 | 124 ± 0.1 |
| 8 | NR4 | Cluster 5 | 0.972 ± 0.03 | 0.013 ± 0.0007 | 215 ± 0.1 | 40 ± 0.03 | 15 ± 0.9 |
| 9 | NR5 | Cluster 2 | 0.295 ± 0.07 | 0.002 ± 0.0004 | 571 ± 0.2 | 165 ± 0.02 | 225 ± 0.09 |
| 10 | NR6 | Cluster 1 | 0.708 ± 0.09 | 0.030 ± 0.0004 | 230 ± 0.2 | 340 ± 0.03 | 150 ± 0.02 |
| 11 | R5 | Cluster 1 | 0.056 ± 0.14 | 0.008 ± 0.0011 | 429 ± 0.03 | 547 ± 0.05 | 140 ± 0.03 |
| 12 | NR7 | Cluster 2 | 0.366 ± 0.4 | 0.020 ± 0.0011 | 888 ± 0.09 | 937 ± 0.09 | 241 ± 0.9 |

*** Experimental label assigned in this study for unbiased interpretation of results. *^ß^* Clinical label assigned in Zabala-Peñafiel et al. 2021 according to patients’ response to Glucantime® treatment. R = Responder; NR= Non-responder. *^+^*Clusters classification according to *in vitro* experimental data gathered in Zabala-Peñafiel et al. 2021. ** Half maximal inhibitory concentration (IC_50_), promastigotes were exposed to serial dilutions of pentavalent (Sb^V^) and trivalent (Sb^III^) antimony for 48 hours as detailed in Zabala-Peñafiel et al. 2021. *** Serine protease activity of total protein extract using Z-FR-AMC [1 mM] as a specific fluorogenic substrate and inhibition assays by incubation with serine proteases inhibitors: PMSF [1 mM], AEBSF [1 mM] and TLCK [100 μM], as detailed in Zabala-Peñafiel et al. 2021
