# Supplementary material for: Novel Insights Into Leishmania (Viannia) braziliensis In Vitro Fitness Guided by Temperature Changes Along With Its Subtilisins and Oligopeptidase B
Source: Front Cell Infect Microbiol. 2022 Apr 21;12:805106. doi: 10.3389/fcimb.2022.805106 (PMC9069558; doi:10.3389/fcimb.2022.805106)
Supplement: Supplementary file 6 [file Table_2.docx]

# Supplementary Table 2. Results of PCR product analysis of *L. (V.) braziliensis* OPB gene using the NCBI BLAST tool

| Isolate* | Target gene product | Sequences with greater similarity index | Max score | Query cover | E value in Blast | Percentage identity |
| --- | --- | --- | --- | --- | --- | --- |
| 4 | OPB | *Leishmania braziliensis* MHOM/BR/75/M2904 genome assembly, chromosome: 9 | 95.3 | 54% | 7x10^-16^ | 95.08% |
| 6 | OPB | *Leishmania braziliensis* MHOM/BR/75/M2904 genome assembly, chromosome: 9 | 89.8 | 39% | 4x10^-14^ | 100% |
| 10 | OPB | *Leishmania braziliensis* MHOM/BR/75/M2904 genome assembly, chromosome: 9 | 86.1 | 51% | 4x10^-13^ | 93.22% |

# *Isolate 4, 6 and 10 were randomly chosen to amplify and sequence OPB gene in order to confirm primers and gene sequences similarity with public data base.
