# Supplementary material for: Novel Insights Into Leishmania (Viannia) braziliensis In Vitro Fitness Guided by Temperature Changes Along With Its Subtilisins and Oligopeptidase B
Source: Front Cell Infect Microbiol. 2022 Apr 21;12:805106. doi: 10.3389/fcimb.2022.805106 (PMC9069558; doi:10.3389/fcimb.2022.805106)
Supplement: Supplementary file 7 [file Table_3.docx]

# Supplementary Table 3. Primers sequences and standard curve parameters for gene expression of *L. (V.) braziliensis* clinical isolates

| Gene target | Primer sequences | Reference | Amplicon length | Slope | Intercept | Coefficient of linearity (r^2^) | Amplification efficiency (%) |
| --- | --- | --- | --- | --- | --- | --- | --- |
| S13 | Fw 5’-GAG CTA ACA CCA GTG GCA CA-3’  Rv 3’-ATC TGG CGA TTT CTC CCT TT-5’ | (Zabala-Peñafiel et al. 2021) | 200 bp | -3.17 | 24.00 | 0.99 | 107.04 |
| S28 | Fw 5’-CAC TGC GCT CCA CAT ACA CT-3’  Rv 3’-GCC TTC ATT CGA GCT ACA GG-5’ | (Zabala-Peñafiel et al. 2021) | 162 bp | -3.21 | 27.00 | 0.98 | 106.04 |
| OPB | Fw 5’-GAG ACT CAG CGA CAG AGA AA-3’  Rev 3’-ATA TCG TTT ATT CTT TCC CAC GC-5’ | (This study) | 122 bp | -3.1 | 21.93 | 0.95 | 110.87 |
| S8 | Fw 5’-GTG CGT AAC GTG AAA GAG CA-3’  Rv 3’-AAG TCG ATG CCG TAA TGC TT-5’ | (Ennes-Vidal et al. 2019; Adaui et al. 2011; Zabala-Peñafiel et al. 2021) | 98 bp | -3.27 | 18.87 | 0.97 | 104.41 |
| Actin | Fw 5’-GTG CGT AAC GTG AAA GAG CA-3’  Rv 3’-GGC AGC TCA AAT GAC TCC TC-5’ | (Ennes-Vidal et al. 2019; Adaui et al. 2011; Zabala-Peñafiel et al. 2021) | 100bp | -3.22 | 19.71 | 0.99 | 105.60 |
