# Supplementary material for: Novel Insights Into Leishmania (Viannia) braziliensis In Vitro Fitness Guided by Temperature Changes Along With Its Subtilisins and Oligopeptidase B
Source: Front Cell Infect Microbiol. 2022 Apr 21;12:805106. doi: 10.3389/fcimb.2022.805106 (PMC9069558; doi:10.3389/fcimb.2022.805106)
Supplement: Supplementary file 8 [file Table_4.docx]

**Supplementary Table 4.** Pearson correlation analysis of *in vitro* data with *L. (V.) braziliensis* clinical isolates.

| Serine proteases genes | *Promastigotes* | *Intracellular amastigotes* | | | |
| --- | --- | --- | --- | --- | --- |
|  | Growth Curve | Infection Index | TNF-α | IL-6 | NO |
| S13 | -0.54^+^ | -0.081 | 0.28** | 0.23** | -0.64 |
| S28 | -0.47^+^ | -0.23 | -0.18 | -0.17 | 0.1* |
| OPB | -0.39 | -0.22 | 0.22** | 0.33** | 0.07* |

Negative Pearson (Ƿ) values between two variables indicate that if one increases the other always decreases; Positive Ƿ values between two variables indicate that if one increases the other always increases: Ƿ = 1.0 to 0.70 (strong correlation***), Ƿ = 0.30 to 0.7 (moderate correlation**), Ƿ = 0 to 0.30 (weak correlation*). ^+^Analysis done with expression in promastigotes data previously published in Zabala-Peñafiel et al 2021.

Results comment: Pearson correlation analysis (Ƿ) measures the correlation degree and its direction, positive or negative, between two variables. The analysis indicated negative correlation from weak (-0.08) to moderate (-0.23) between each gene expression data and infection index. Additionally, positive correlation was seen between S13 gene expression and TNF-α (0.28) and IL-6 (0.23) production, similarly, between OPB gene expression and TNF-α (0.22) and IL-6 (0.33). On the contrary, negative correlation values were seen between S28 gene expression and TNF-α (-0.18) and IL-6 (-0.17)
